# Can vaccination coverage be improved by reducing missed opportunities for vaccination? Findings from assessments in Chad and Malawi using the new WHO methodology

**DOI:** 10.1371/journal.pone.0210648

**Published:** 2019-01-24

**Authors:** Ikechukwu Udo Ogbuanu, Anyie J. Li, Blanche-philomene Melanga Anya, Mbaihol Tamadji, Geoffrey Chirwa, Kwame W. Chiwaya, Mohamed El-Hafiz Djalal, Dah Cheikh, Zorodzai Machekanyanga, Joseph Okeibunor, Colin Sanderson, Richard Mihigo

**Affiliations:** 1 World Health Organization, Headquarters, Geneva, Switzerland; 2 ASPPH/CDC Allan Rosenfield Global Health Fellowship and PHI/CDC Global Health Fellowship, Atlanta, Georgia, United States of America; 3 World Health Organization, African Regional Office, Brazzaville, Republic of Congo; 4 Le Ministère de la Santé Publique de la République du Tchad, N’Djamena, Chad; 5 Ministry of Health, Lilongwe, Malawi; 6 World Health Organization, Country Office, Lilongwe, Malawi; 7 World Health Organization, Country Office, N’Djamena, Chad; 8 World Health Organization, Central Africa Inter-Country Support Team (IST), Libreville, Gabon; 9 World Health Organization, East and Southern Africa Inter-Country Support Team (IST), Harare, Zimbabwe; 10 London School of Hygiene and Tropical Medicine, London, United Kingdom; The University of Warwick, UNITED KINGDOM

## Abstract

**Background:**

In 2015, the World Health Organization (WHO) updated the global methodology for assessing and reducing missed opportunities for vaccination (MOV), when eligible children have contact with the health system but are not vaccinated. This paper presents the results of two pilot assessments conducted in Chad and Malawi.

**Methods:**

Using the ten-step global WHO MOV strategy, we purposively selected districts and health facilities, with non-probabilistic sampling of <24 month old children for exit interviews of caregivers and self-administered knowledge, attitudes, and practices (KAP) surveys of health workers. MOV were calculated based on a child’s documented vaccination history (i.e., from a home-based record (HBR) or a health facility vaccination register), including selected vaccines in the national schedule.

**Results:**

Respondents included caregivers of 353 children in Chad and of 580 children in Malawi. Among those with documented vaccination history, 82% (195/238) were eligible for vaccination in Chad and 47% (225/483) in Malawi. Among eligible children, 51% (99/195) in Chad, and 66% (149/225) in Malawi had one or more MOV on the survey date. During non-vaccination visits, 77% (24/31) of children eligible for vaccination in Chad and 92% (119/129) in Malawi had a MOV compared to 46% (75/164) and 31% (30/96) during vaccination visits, respectively. Among health workers, 92% in Chad and 88% in Malawi were unable to correctly identify valid contraindications for vaccination.

**Conclusion:**

The new MOV tool was able to characterize the type and potential causes of MOV. In both countries, the findings of the assessments point to two major barriers to full vaccination of eligible children—a lack of coordination between vaccination and curative health services and incomplete vaccination during vaccination visits. National immunization programs should explore tailored efforts to improve health worker practices and to increase vaccine delivery by making better use of existing health service contacts.

## Introduction

Since it was established in 1974, the Expanded Programme on Immunization (EPI) has contributed to improvements in child health and survival globally [1–3]. Despite progress in the control of vaccine-preventable diseases and implementation of the Global Vaccine Action Plan 2011–2020 (GVAP), between 2012 and 2015, coverage with the third dose of diphtheria-tetanus-pertussis vaccine (DTP) in the African Region of the World Health Organization (WHO) has stalled at around 70% [4–7]. This persistence of low coverage is attributable to many factors, among which are missed opportunities for vaccination (MOV) [8].

A MOV includes any contact with health services by a child (or adult) who is eligible for vaccination (unvaccinated or partially vaccinated/not up-to-date, and free of contraindications), but which does not result in the individual receiving all the vaccine doses for which he or she is eligible [9, 10]. The first systematic literature review of MOV conducted in 1993 found a median global MOV prevalence of 67% among the subpopulation of children and women who were eligible for vaccination at the time of contact [9].

Studies of MOV have been conducted in many countries using different methodologies [11–21]. However, to respond to the need for a coordinated strategy, WHO has drafted an updated and standardized global methodology for countries to assess and reduce MOV, in collaboration with multiple immunization partners [10]. The WHO MOV strategy builds on the 2013 Pan American Health Organization (PAHO) protocol, but differs from it by limiting sampling to children aged 0–23 months, simplifying the health facility sampling strategy, incorporating qualitative methodologies, and emphasizing implementation of interventions and a follow-up component [22]. As a result, the assessment field work now concludes with the participation of all local immunization partners in an intervention brainstorming session. The brainstorming session aims to synthesize all the available preliminary data and to build consensus and advocacy for an endorsed and funded work plan to reduce MOVs, as part of a broader goal to strengthen health systems and immunization programmes.

Field work using the new MOV methodology has now been completed in 11 countries across four of the six WHO regions (African, South East Asian, Eastern Mediterranean and Western Pacific regions). In order to ensure that this global methodology is relevant to the African context, and to help institutionalize efforts to reduce MOV, in 2015 the WHO Regional Office for Africa, with technical support from WHO headquarters, piloted this methodology in Chad and Malawi [23]. The objective of this paper is to document the experiences and lessons learned in Chad and Malawi for future countries in other regions who may be interested in using the MOV strategy to address persistent vaccination coverage gaps.

## Methods

### Study design

**Fig 1:**
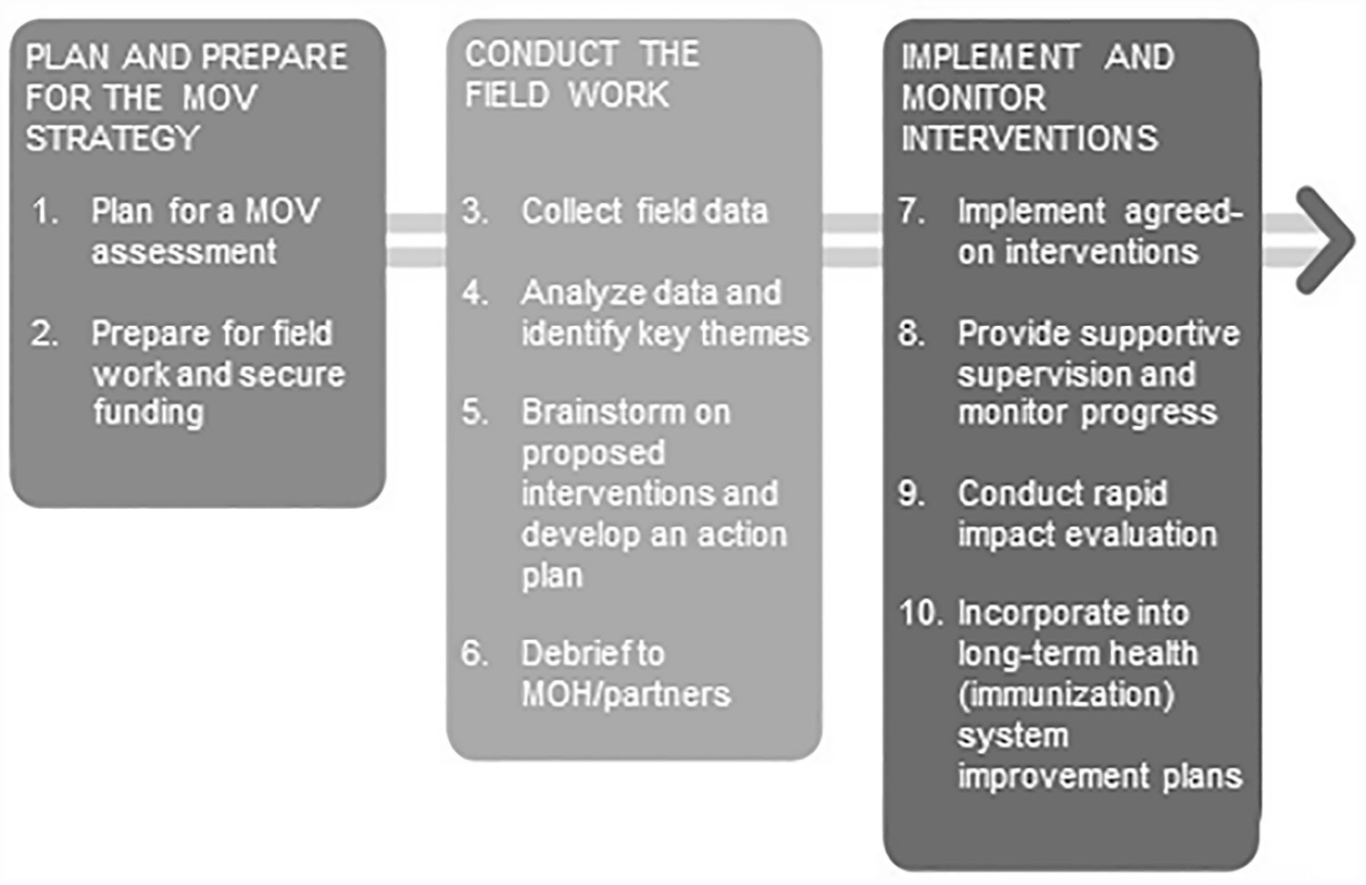
The ten steps of the updated WHO strategy to reduce missed opportunities for vaccination (MOV).

This study was based on the new MOV strategy as detailed in the WHO *Planning Guide to Reduce Missed Opportunities for Vaccination (MOV)* and *Methodology for the Assessment of Missed Opportunities for Vaccination* [10, 24, 25]. The MOV strategy is a ten-step mixed-methods approach that triangulates quantitative and qualitative data from a broad range of interview sources including caregivers, health workers, and healthcare administrators and managers (Fig 1). The assessment aims to answer three key questions: (1) how many opportunities are being missed, (2) why are these opportunities being missed, and (3) what can be done to reduce MOV?

The quantitative arm of the methodology consists of voluntary exit surveys with caregivers and anonymous self-administered health worker knowledge, attitudes and practices (KAP) surveys. The qualitative arm collects information from cross-sectional samples, using focus group discussions (with caregivers and health workers) and in-depth interviews (with healthcare administrators). Quantitative results from Chad and Malawi are presented in this paper.

### Data collection instruments

Prior to data collection, the generic caregiver exit surveys and health worker surveys were adapted to the country contexts of Chad and Malawi (S1–S4 Files) [10]. Exit surveys covered vaccination history, awareness of routine immunization services, and quality of vaccination services. Health worker surveys covered knowledge, attitudes, and practices of vaccination with an additional section on immunization practices and decision making for health workers who routinely administer vaccines.

### Sample size and participant selection

The new WHO methodology recommends a simplified quota sampling strategy, as the results are not intended to be nationally representative. It utilizes purposive selection of districts and health facilities to represent various geographic regions and performance levels based on coverage of the third dose of DTP-hepatitis B-*Haemophilus influenzae* type b, or pentavalent, vaccine. An effort was made to include public, private, and non-governmental organization (NGO) facilities of varying sizes in both rural and urban settings.

In Chad, the sample included six districts and five health facilities per district. Due to security concerns at the time, data collection was limited to districts in two regions (N’Djamena and Chari Baguirmi). Data collection in Malawi included ten districts selected from all three regions of the country (North, South, and Central) with three health facilities per district.

The primary units of analysis were children aged 0–23 months who attended the selected health facilities for any type of service on the day of the assessment. Given variations in availability of home-based records (HBRs) in different countries, the new methodology recommends a target sample size of 600 eligible children and 300 health workers. Each data collection team was assigned to complete 20 exit surveys and 10 health worker KAP surveys per facility. However, because health facilities were of differing sizes, teams continued to survey caregivers and health workers for the entire duration of their visit to the health facility (one day), even if the health facility quota had been met, to compensate for lower patient volume in smaller facilities on subsequent days.

Children were eligible for this study if they were accompanied by a caregiver over the age of 15 years. If a caregiver was accompanied by more than one age-eligible child, the youngest child was selected for the survey. To assess coordination of immunization delivery within health facilities, all available health workers were eligible for the KAP survey, regardless of their involvement in routine immunization service delivery. However, only health workers who routinely administered vaccines completed the additional section on immunization practices and decision-making.

### Data collection

In 2015, field staff in both Chad and Malawi were trained during the three days immediately preceding field work. Particular attention was paid to the use of the tablet survey software platform (*Zegeba AS* [Alesund, Norway]). All data were collected electronically *ab initio* in Malawi. In Chad, due to a limited number of available tablets, data collection teams of two shared one tablet, resulting in paper-based data collection for approximately half of the surveys. These data were later entered into *Zegeba*. Field work was undertaken in Chad during July 16–20 and in Malawi during August 13–18.

Field team assignments ensured an appropriate mix of local language proficiency and professional background. Data collectors positioned themselves at the exits of each selected health facility. They approached caregivers who were accompanied by a child 0–23 months of age as they were exiting the facility and asked if they were willing to participate in the survey. Each caregiver was asked for their child’s HBR, from which the vaccination dates were recorded. If the HBR was present, this was the sole source of vaccination dates. If no HBR was available, the data collector noted the child’s demographic data on a separate form. At the end of the exit interviews, the demographic data were used to search for the child’s vaccination data in the health facility vaccination registers, which were paper-based at all health facilities surveyed. Only registers from the current health facility were searched. If the caregiver did not have the child’s HBR and the data collector was not able to find the child’s record in the health facility vaccination register, no vaccination dates were recorded. No verbal report of vaccination dates was accepted. In Chad, “vaccination card” is the terminology used for the written vaccination record kept by caregivers, while “health passport” is the terminology used in Malawi. Throughout this paper, “HBR” is used to refer to the written vaccination record kept by caregivers in both countries. Photos of the HBR and health facility vaccination registers were taken for subsequent data validation and cleaning.

During the exit interviews, caregivers were asked their primary reason for bringing their child to the health care facility. Based on their response, the child’s visit was categorized as: 1) medical consultation (child is sick), 2) vaccination visit, 3) healthy child visit or developmental check-up, 4) child accompanying adult (not for treatment or vaccination of the child), 5) hospitalization (child was admitted or is still on admission), or 6) other (specific reason recorded). Exit interviews lasted approximately 20 minutes.

Health workers completed a self-administered KAP survey. Health worker KAP surveys were either self-administered on paper and subsequently entered into the tablets or completed on a tablet with a surveyor available to help work through the electronic platform as needed. KAP surveys lasted approximately 30 minutes.

All tablets were password-protected and field teams only had access to data from their assigned field site. Only key study staff had access to all surveys. All data were routinely uploaded and backed up to a secure network. Paper survey data were entered nightly into the electronic platform and paper versions were securely stored at the WHO country office.

### Data analysis

**Fig 2:**
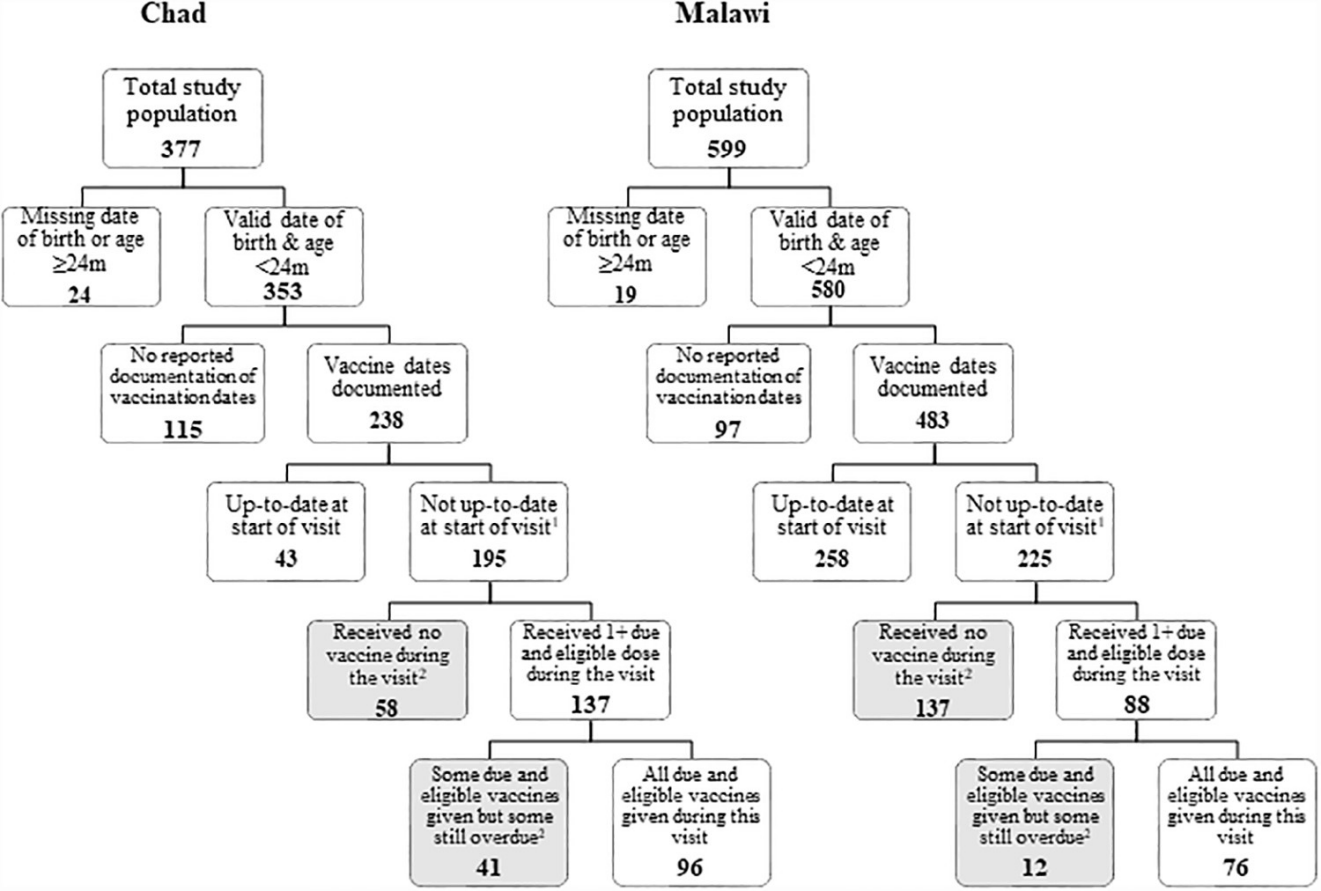
Health-facility-based flow-chart for determining missed opportunities for vaccination (MOV), Chad and Malawi, 2015. ^1^All children were without valid contraindications and had 1+ eligible dose due. ^2^Missed opportunity for vaccination (MOV): contact with health services by a child (or adult) who is eligible for vaccination (unvaccinated, partially vaccinated, or not up-to-date, and free of contraindications to vaccination), which does not result in the individual receiving all the vaccine doses for which he or she is eligible [9, 10].

Data were analyzed using STATA (version 14.2, College Station, Texas). We produced frequency distributions for each variable to explore themes within the caregiver exit and health worker KAP surveys. To align our analysis with previous studies, we produced a flow-chart for identifying MOV (Fig 2) [9]. A MOV was determined based on the child’s age on the interview date, eligibility for various vaccines (according to the national schedule), and presence of potential contraindications (as reported by the caregiver). Only children with either documented evidence of vaccination dates or a blank vaccination card (indicating no vaccines had been given) and who were eligible for vaccination were included in the calculation of MOV. All antigens in the country’s national immunization program were used in determining MOV in both countries except for yellow fever vaccine in Chad (as it was not available in every district at the time of the assessment). As a health service-based assessment, we calculated the prevalence of MOV among children in need of immunization, excluding those already up-to-date or with valid contraindications. This estimate of MOV measures the inefficiencies of health services [9]. MOV were further cross-tabulated by reason for visit and child and caregiver factors.

**Fig 3:**
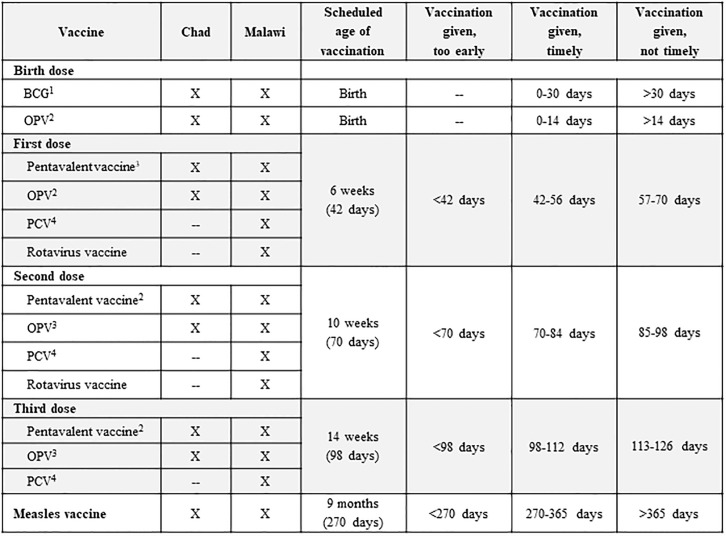
Time intervals used for classifying timeliness of vaccination doses received by surveyed children, using the nationally recommended ages for vaccination: Chad and Malawi, 2015. ^1^bacille Calmette-Guerin (BCG) vaccine. ^2^Oral poliovirus vaccine (OPV). ^3^Diphtheria-tetanus-pertussis-hepatitis B-*Haemophilus influenzae* type b vaccine (pentavalent) vaccine. ^4^Pneumococcal conjugate vaccine (PCV).

We determined timeliness and age intervals for early, timely and not timely vaccination using documented birth and vaccination dates. (Fig 3). These categories allowed for grace periods based on the countries’ national policies and previous timeliness studies in Africa [26, 27].

### Ethical approval

In Chad, the study protocol was assessed by the Ministry of Health (MOH) and was deemed a program review. As such, it was not subjected to further review by the Institutional Review Board (IRB). In Malawi, the study protocol was submitted to the Malawi MOH IRB (National Health Sciences Research Committee) for review prior to data collection and was also considered exempt as a public health program assessment. As a public health program assessment, written consent was not required. However, in both countries, data collectors obtained verbal consent from all caregivers and health workers prior to administering the surveys. The verbal consent procedure was as approved by the respective ethics committees and each response was recorded on the questionnaires.

## Results

There were a total of 377 completed exit interviews of caregivers and 179 health worker surveys in Chad; 353 children met eligibility criteria and were included in the analysis, and 238 (67%) had documented vaccination histories (Tables 1 and 2; Fig 2). In Malawi, there were 599 completed exit surveys of caregivers and 262 health worker surveys; 580 children met eligibility criteria and were included in the analysis; 483 (83%) had documented vaccination histories (Tables 1 and 2; Fig 2). Results presented here only account for children with documented vaccination dates. None of the children with documented vaccination histories reported any contraindications. The median number of exit interviews conducted per facility was 9 in Chad (interquartile range (IQR), 5–17) and 19 in Malawi (IQR, 17–23). The median number of health worker KAP surveys per facility was 10 in Malawi (IQR, 7–10). Health worker KAP surveys in Chad did not record information on location of the health facilities. There was a high response rate in both Chad and Malawi with only six and three refusals among caregivers, respectively. There were no refusals among health workers in either country. MOV field data collection in both countries was completed in three days.

**Table 1:** Characteristics of surveyed caregivers of children with documented vaccination dates: Chad and Malawi, 2015.

		Chad		Malawi	
		n	%	n	%
**CAREGIVER SURVEY**	**TOTAL**	**238**		**483**	
***Child demographics***					
**Sex**		**197**		**476**	
Male		77	39	214	45
Female		120	61	262	55
**Age**		**238**		**483**	
<12 months		230	97	335	69
≥12 months		8	3	148	31
**Ever vaccinated**		**226**		**461**	
Yes		206	91	424	92
No		20	9	37	8
***Caregiver demographics***					
**Sex**		**234**		**470**	
Male		8	3	5	1
Female		226	97	465	99
**Relationship to child**		**206**		**465**	
Mother		190	92	460	99
Father		6	3	2	<1
Other (e.g., grandparent, uncle/aunt, sibling)		10	5	3	1
**Educational Level**		**229**		**474**	
None		111	48	98	21
At least some primary		26	11	267	56
At least some secondary		92	40	109	23
***Health facility visit***					
**Type of Health Facility**		**238**		**483**	
Public or government		193	81	471	98
Private		28	12	1	<1
Other (NGO^1^ faith-based, etc.)		17	7	11	2
**Reason for visit**		**238**		**483**	
Medical consultation^2^		21	9	223	46
Vaccination		194	82	131	27
Healthy child visit or developmental check-up		--	--	53	11
Child is accompanying adult^3^		5	2	39	8
Other or no reason reported		18		37	8
**Child has home-based record**		**210**		**461**	
Yes, available at visit		196	93	457	99
Yes, but not available at visit		12	6	2	<1
No		2	1	2	<1
**Did staff ask for the card?**		**210**		**465**	
Yes		180	86	292	63
No		23	11	173	37
No, but they asked about vaccines given		7	3	0	0

^1^ Non-governmental organization (NGO)

^2^ Child is sick, injured, or other medical consultation

^3^ Child is accompanying an adult or sibling (not visiting for treatment of vaccination)

**Table 2:** Characteristics and knowledge, attitudes, and practices of surveyed health workers: Chad and Malawi, 2015.

		Chad		Malawi	
		n	%	n	%
**HEALTH WORKER SURVEY**	**TOTAL**	**179**		**262**	
***Health worker demographics***					
**Sex**		**168**		**257**	
Male		63	38	148	58
Female		105	63	109	42
**Professional Training**		**171**		**261**	
Clinician		10	6	25	10
Nurse/Midwife		124	73	40	15
Nursing Assistant (Chad)/Assistant Environmental Health Officer (Malawi)		15	9	5	2
Community Health Worker (Chad)/Health Surveillance Assistant (Malawi)		5	3	172	66
Other		17	10	19	7
**Type of Service**		**166**		**262**	
Public or government		120	72	256	98
Private		22	13	1	<1
Other		24	15	5	2
**Ever trained in vaccination or vaccine-preventable diseases**		**167**		**256**	
Yes		112	67	187	73
No		55	33	69	27
***Health worker knowledge*, *attitudes*, *practices***					
**I feel my knowledge of vaccination is insufficient or out of date**		**156**		**253**	
Agree		67	43	118	47
Disagree		89	57	135	53
**What are contraindications for any vaccine?**		**150**		**250**	
Local reaction to previous dose		23	15	23	9
Low grade fever		52	35	123	49
Seizures under medical treatment		8	5	24	10
Pneumonia and other serious diseases		10	8	30	12
None of the above		57	38	50	20
**When should vaccination status be assessed?**		**148**		**253**	
Child’s wellness/routine visit		100	68	18	7
Consultation for any illness		19	13	52	21
When a child is accompanying an adult for any reason		5	3	45	18
All of the above		24	16	138	55
**What instructions do you give caregivers when you give them a new HBR?**^1^		**142**		**243**	
Keep this card safe		81	57	166	68
Bring this card to all visits to the health facility		94	66	228	94
Bring this card only when you come for vaccinations		58	41	37	15
No instructions given		5	4	4	2
**There is sufficient staff offering immunization services at this facility**		**159**		**243**	
Agree		86	54	141	58
Disagree		73	46	102	42
**There is enough vaccine supply (vials) for all patients in need**		**153**		**235**	
Agree		102	67	156	66
Disagree		51	33	79	34

^1^ Respondent allowed to select multiple responses

### Caregiver exit interviews

#### Demographics

Among children with documented vaccination dates, the majority of respondents (84%) in Chad were clustered within four districts around the capital city of N’Djamena (data available according to PLOS ONE’s Data Availability policy). In Malawi, the respondents were evenly spread over the selected districts across all three regions, with each district accounting for 5–15% of the total sample size. In both countries, the majority of interviews were conducted in public or government facilities (81% in Chad and 98% in Malawi) (Table 1). Other socio-demographic characteristics are shown in Table 1.

#### Vaccination

In Chad, among the 238 children with documented vaccination dates, 196 (93%) of the 210 caregivers who responded to the HBR question had their child’s HBR available on the day of the study (Table 1). In Malawi, among the 483 with documented dates, 457 (99%) of the 461 who responded had the HBR. During the service encounter, 86% of caregivers of children with documented vaccination dates in Chad and 63% in Malawi reported that the health workers had asked to review the HBR.

Prior to the visit, almost all of the sampled children with documented vaccination dates (91% in Chad and 92% in Malawi) had received at least one previous vaccination (Table 1). However, prior to the visit, 82% (195/238) of those surveyed in Chad and 47% (225/483) in Malawi, respectively, were still under-vaccinated (missing at least one dose for which they were age-eligible) (Table 3).

**Table 3:** Missed opportunities for vaccination (MOV)^1^ by reason for visit, Chad and Malawi, 2015.

					Child-based MOV		Dose-based MOV^2^		
	Total children with documented vaccination dates	No. of children with 1+ eligible doses due			Proportion of eligible children with 1+ MOV		No. of eligible doses among children with 1+ eligible doses due	Proportion of total eligible doses missed	
	n	n	%		n	%	n	n	%
**Chad**									
Vaccination visit	194	164	85		75	46	314	106	34
*Non-vaccination visit*									
Medical consultation	21	13	62		12	92	25	23	92
Healthy child visit or check-up	--	--	--		--	--	--	--	--
Child is accompanying adult	5	3	60		3	100	7	7	100
Other	7	6	86		5	83	9	8	89
No reason reported	11	9	82		4	44	17	5	29
*Non-vaccination visit total*	*44*	*31*	*70*		*24*	*77*	*58*	*43*	*74*
**Total**	**238**	**195**	**82**		**99**	**51**	**372**	**149**	**40**
**Malawi**									
Vaccination visit	131	96	73		30	31	253	59	23
*Non-vaccination visit*									
Medical consultation	223	83	37		80	96	165	159	96
Healthy child visit or check-up	53	13	25		13	100	20	19	95
Child is accompanying adult	39	18	46		16	89	37	34	92
Other	28	11	39		8	73	20	13	65
No reason reported	9	4	44		2	50	11	5	45
*Non-vaccination visit total*	*352*	*129*	*37*		*119*	*92*	*253*	*230*	*91*
**Total**	**483**	**225**	**47**		**149**	**66**	**506**	**289**	**57**

^1^Missed opportunity for vaccination (MOV): contact with health services by a child (or adult) who is eligible for vaccination (unvaccinated, partially vaccinated, or not up-to-date, and free of contraindications to vaccination), which does not result in the individual receiving all the vaccine doses for which he or she is eligible [9,10]

^2^MOV based on the total number of vaccine doses children are eligible for and administered per type of visit

#### Missed opportunities for vaccination (MOV)

Of the children eligible for at least one vaccination, 51% in Chad and 66% in Malawi had a MOV at the end of the visit (i.e. did not receive at least one of the vaccines for which they were eligible) (Table 3). In Chad and Malawi, most (77% and 92% respectively) children visiting for a non-vaccination visit and eligible for vaccination had a MOV. Among those who visited the facility specifically for vaccination, 46% and 31% respectively still had a MOV. Among the total number of vaccine doses children were eligible for, 40% of doses in Chad and 57% in Malawi were missed.

In Chad, 49% of children under 12 months had a MOV compared with six of the seven children over 12 months (86%) (Table 4). In Malawi, MOV occurrence was also higher (94%) among children over 12 months (33/35) when compared to children <12 months (61%; 116/190).

**Table 4:** Missed opportunities for vaccination (MOV)^1^ among children with 1+ eligible doses due stratified by child and caregiver factors: Chad and Malawi, 2015.

	Chad			Malawi		
	Total^2^	MOV (n)	%	Total^2^	MOV (n)	%
**Total**	**195**			**225**		
**Sex of child**	**160**			**223**		
Male	62	33	53	101	66	65
Female	98	51	52	122	82	67
**Age of child**	**195**			**225**		
<12 months	188	93	49	190	116	61
≥12 months	7	6	86	35	33	94
**Caregiver educational level**	**188**			**221**		
At least some secondary	72	33	46	50	33	66
At least some primary	23	10	43	122	81	66
None	93	52	56	49	33	67
**Travel time to facility**	**183**			**221**		
<30 minutes	125	63	50	41	24	59
30–59 minutes	37	18	49	39	26	67
1 hour or more	21	11	52	141	97	69
**Type of facility**	**195**			**225**		
Public	159	79	50	215	139	65
Private	22	12	55	1	1	100
NGO	14	8	57	3	3	100
Faith-based	--	--	--	6	6	100
**Vaccination card at appointment**	**195**			**225**		
Yes, and I have it with me	164	80	49	211	140	66
Yes, but I do not have it with me	6	6	100	1	1	100
No	1	0	0	13	8	62
No Response	24	13	54	--	--	--

^1^ Missed opportunity for vaccination (MOV): contact with health services by a child (or adult) who is eligible for vaccination (unvaccinated, partially vaccinated, or not up-to-date, and free of contraindications to vaccination), which does not result in the individual receiving all the vaccine doses for which he or she is eligible [9, 10]

^2^ Children with documented vaccination dates and eligible for one or more vaccine doses

#### Timeliness of vaccination

The percentage of children receiving their vaccines on time was lower for vaccines scheduled at older ages (Table 5). In Chad, the percentage of children that received pentavalent vaccine within 14 days of the recommended age decreased 32 percentage points from the first dose to the third dose and in Malawi, 28 percentage points. However, timeliness of vaccines given at nine months of age (measles in Chad and Malawi) was high in both countries (Table 5).

**Table 5:** Timeliness of vaccine doses administered to surveyed children with documented vaccination history by vaccine: Chad and Malawi, 2015.

Vaccine dose	Chad				Malawi			
	Total number of children who received dose	Timeliness^1^			Total number of children who received dose	Timeliness^1^		
		Too early (%)	Timely (%)	Not timely (%)		Too early (%)	Timely (%)	Not timely (%)
**Birth dose**								
BCG^2^	213	--	**74**	25	420	--	**83**	17
OPV^3^	170	--	**54**	46	306	--	**84**	15
**First dose**								
Pentavalent vaccine^4^	149	14	**55**	31	373	9	**52**	39
OPV^3^	159	11	**57**	32	379	8	**49**	42
PCV^5^	--	--	**—**	--	374	9	**52**	39
Rotavirus vaccine	--	--	**—**	--	374	9	**51**	40
**Second dose**								
Pentavalent vaccine^3^	97	10	**38**	52	333	6	**36**	58
OPV^4^	99	8	**37**	55	333	8	**32**	61
PCV^5^	--	--	**—**	--	330	7	**35**	59
Rotavirus vaccine	--	--	**—**	--	326	5	**37**	58
**Third dose**								
Pentavalent vaccine^3^	62	15	**23**	63	289	5	**24**	72
OPV^4^	60	10	**25**	65	280	7	**20**	73
PCV^5^	--	--	**—**	--	275	5	**24**	70
**Measles vaccine**	39	28	**69**	3	157	15	**78**	7

^1^Please see Fig 3 for cut-offs and Chad and Malawi Expanded Programme on Immunization schedules used for this analysis

^2^ bacille Calmette-Guerin (BCG) vaccine

^3^ Diphtheria-tetanus-pertussis-hepatitis B-*Haemophilus influenzae* type b vaccine (pentavalent)

^4^ Oral poliovirus vaccine (OPV)

### Health worker KAP survey

#### Demographics and training

Among the health workers interviewed, the majority in Chad were nurses or midwives (73%), but in Malawi the majority were health surveillance assistants (HSAs; 66%) (Table 2). HSAs are the only health worker cadre that administer vaccines in Malawi. Following their pre-service training, over one-quarter of all health workers interviewed (33% in Chad and 27% in Malawi) had not subsequently received on-the-job training on vaccination or vaccine preventable diseases. Of those who had been trained, 69% in Chad had received that training within the last two years. In Malawi, over half (55%) received their last training more than four years prior to the survey.

#### Knowledge, attitudes, and practices

Almost half of health workers in both countries felt their knowledge of vaccination was insufficient or out of date (43% in Chad and 47% in Malawi) (Table 2). Half of health workers (49%) in Malawi incorrectly identified low-grade fever as a contraindication for any vaccination.

In Chad, 68% of the respondents indicated that vaccination status should only be assessed at a wellness visit (Table 2). On the other hand, over half (55%) of the health workers in Malawi reported that vaccination status should be assessed at several points of contact with the healthcare system. Such contacts include a wellness visit, consultation for any illness, or when a child is accompanying an adult for any reason.

In both countries, one-third (33% in Chad and 34% in Malawi) of the health workers felt they did not have enough vaccine supply for all the children seeking vaccination services on the day of the survey (Table 2). Additionally, only a little over half felt that there was sufficient staff offering immunization services at their facility (54% in Chad and 58% in Malawi).

## Discussion

In both Chad and Malawi, the first two countries to pilot the new WHO MOV methodology, we were able to characterize the type and potential causes of MOV that occurred. We identified significant opportunities to improve efficiencies by standardizing catch-up policies and vaccination checks, addressing health worker constraints and improving knowledge surrounding vaccination schedules and contraindications, and ensuring necessary vaccination supplies are available.

In both countries, there was high uptake of vaccination services, as indicated by the large proportion of children that had previously received at least one dose of a vaccine. However, many children are still not receiving all the vaccines they are eligible for, even during vaccination visits. Given that the percentage of children already up-to-date prior to the visit was low in both countries (18% in Chad and 53% in Malawi), a large proportion of children visiting health facilities at any given time are likely to be eligible for vaccine doses. Although many caregivers reported that the health worker had asked to see their child’s HBR, low percentages of health workers in both countries reported sufficient knowledge of vaccination and low percentages reported that vaccination status should be checked at every visit. Therefore, health workers may be asking for the HBR, but may not be reviewing the HBRs for vaccination eligibility, and instead using it largely to record or verify demographic data. Interventions such as conducting vaccination status checks at all visits, especially if the process or policy is incorporated into a system improvement plan, can result in increases in both timeliness and overall vaccination coverage. The results show that status checks can become more important as children get older, since the proportion of children with MOV increased with age in both countries.

The data we have presented also support increased coordination of vaccination with curative health services. Over three-quarters of children at non-vaccination visits missed opportunities to be vaccinated; it is therefore feasible to increase immunization coverage simply by making better use of existing health service contacts. Standard clinical practice should include vaccination status checks at every health service encounter. Additionally, caregivers need to be encouraged to retain and bring the HBR to every health service encounter. Availability and use of the HBR remains a challenge in many countries in the African Region [28]. HBRs have the dual function of serving as a reminder to caregivers of the vaccination schedule, and as a means of communication between the health worker and the caregiver [29]. While there was high HBR availability in both countries among children with documented vaccination dates, there was a notable difference in the total number of children with documented vaccination dates (67% in Chad and 83% in Malawi; Fig 2). In Malawi, the HBR contains records of other maternal and child health services, in addition to vaccination records, and is required at every health service encounter; this could account for the higher number of children with documented vaccination dates in Malawi, as dates are more easily accessible through the HBR than a health facility register.

Health workers must be given the appropriate tools and resources to address vaccination gaps. In order to reduce MOV and increase vaccination coverage in these countries, it is imperative that health workers are equipped to properly review HBRs and provide catch-up vaccination to eligible children. To ensure synergy between vaccination and other services, all health workers, not only immunizing staff, must be able to correctly review a HBR for eligibility. Additionally, the issue of human resource constraints emerged from the results, with over 40% of health workers in both countries indicating that there was insufficient staff offering immunization services at their facility. Health facilities should consider leveraging other health workers (such as administrative and security staff) to assist with non-clinical aspects of vaccination services, such as instituting a “triage” and “exit” station where vaccination status can be checked and recorded. This will help to alleviate some of the pressure on the immunization staff, allowing them to spend more time on administering the needed vaccines and on inter-personal communication. Where possible, national or subnational ministries of health should consider a functional review of the human resources available in the health system and consider re-alignment of staff or hiring more staff to fill needed roles, as appropriate.

Finally, opportunities for vaccination continue to be missed because of a lack of needed vaccines and other supplies to vaccinate all eligible children. Both countries must work to ensure a constant and sufficient supply of vaccines and vaccination-related materials down to the lowest levels, so that all eligible children have access to vaccination services whenever they visit health facilities. Countries should institutionalize regular monitoring of stock levels and facilitate a mechanism to redistribute stocks as needed.

### Brainstorming session to develop a local intervention plan to reduce MOV

As part of the field work, the new WHO MOV methodology prescribes a process to develop an action plan to reduce MOV. Following the data collection phase, *Step 5* of the new WHO methodology outlines the process for a two-day brain storming session to accomplish this (Fig 1) [10, 24, 25]. To ensure funding and sustainability, it is recommended that the MOV action plan be endorsed by key decision makers, top ministry officials, immunization partners and other stakeholders within the country (*Step 6*) [10, 24, 25].

Following the debriefing of results and consensus-building in Chad and Malawi, WHO and other global partners are currently working with in-country partners on efforts to address MOV, utilizing a variety of approaches as outlined in the respective country action plans. In Chad, a multi-pronged approach is being implemented, focusing on innovative health worker trainings (by including non-immunization staff for the first time and incorporating a MOV supportive supervision plan) and system changes at the facility-level (supported through a directive signed by the Minister of Health) (M. Djalal, personal communication, October 2017). One of the innovative approaches that has been implemented in Chad was the distribution of “vaccination tokens” to all *curative* services at health centers in the implementation districts. The curative health staff were trained to screen all eligible children for vaccination status and to record any missing doses within the tokens. The curative health staff then referred the eligible children to the vaccination service area (EPI clinic). At the EPI clinic, the vaccinators retrieved the tokens from the child’s caregiver, administered the missing doses and retained the tokens. Preliminary data show an overall 84% retrieval rate of the tokens and a 12% increase in the number of doses administered in 2017, compared with the same months in the previous year (2016) (monthly immunization data reported to AFRO, as presented to SAGE, October 2017 [30] and M. Djalal, personal communication, October 2017). Further evaluation of the impact of the token system will be reported in a subsequent publication.

In Malawi, in addition to interventions targeting health workers and system-level changes, there has also been a focus on addressing knowledge and information gaps among caregivers (including reviving health talks for parents in the waiting room and use of community health workers for education) (G. Chirwa, personal communication, February 2018). Malawi is also focusing on alleviating logistical issues related to supplies of vaccines and vaccination materials. In both countries, post intervention impact evaluations are being planned. Efforts to ensure sustainability include incorporating MOV activities into annual EPI work plans, partnering with other in-country immunization stakeholders, and requesting (or aligning activities with) long-term support through funders such as Gavi, the Vaccine Alliance.

### Limitations

The assessment results we have reported are not without limitations. Because this was a pilot, the questionnaire tools were still being tested. As a result, certain questions regarding health worker knowledge were indicated as single-choice options, where multiple-choice options were warranted, causing potentially biased results. Additionally, due to the non-random sampling strategy, the results of these assessments are not intended to be nationally representative. The results we have presented should be viewed as a programmatic assessment whose intent is to diagnose major program issues and implement actions to reduce MOV.

Due to the per-protocol sampling of only health facilities, the methodology does not account for MOV during outreach services or within communities. However, the resulting improvements in health worker and caregiver practices are expected to have a positive spill-over effect on the catchment communities. In addition, by limiting the estimation of MOV to attendees of health facilities with retrievable recorded vaccination dates (HBR or health facility vaccination register) or a verified blank vaccination card for accuracy, our estimates of MOV are likely biased towards the null. Many of the children who were excluded in our analysis are likely to have had more MOV, and therefore the true impact of reducing MOV is possibly much higher than we have estimated.

Finally, the cross-sectional methodology is useful for establishing factors *associated* with MOV. However, these associations may not be causal; interventions designed to reduce them may therefore not eliminate the associated MOV.

### Conclusion

The first field experiences with using the new WHO MOV methodology have shown that the proposed methodology provides a breadth of actionable information, while focusing in on the primary reasons for MOV. The new methodology was less complex to implement than a nationally representative survey. MOV field data collection in both countries was completed in only three days and preliminary data for brainstorming on potential interventions were available one day after field data collection. These suggest that the new methodology is implementable in resource-limited settings in Africa and other regions.

The high proportions of visits with MOV in Chad and Malawi suggest that interventions to reduce MOV in health service settings may be a potential quick win for improving coverage and equity in these settings. The findings and proposed interventions in both countries illustrate the multi-faceted approach needed to resolve bottlenecks; our results revealed both supply and demand-side barriers to fully immunizing children who already have access to health services. As interventions are implemented in more countries, the WHO methodology will be updated to include guidance for monitoring and evaluation of impact.

Lessons learned from these two country studies include the use of the field work process as an advocacy tool for high-level visibility of the issues raised; the focus on tailored, simple interventions; the critical role (but absence) of sustainable supportive supervision in both countries; the need to capitalize on existing platforms and build synergies with other partners; and an awareness of the elements needed for successful implementation of interventions, including innovative thinking, working with multiple partners, collaborations with *non-immunization* MOH staff, and ongoing accountability for results.

Finally, the new methodology lends itself to adaptation according to country needs and available resources. For example, a local immunization program may already have evidence that MOV contributes to low immunization coverage (either from previous program reviews, coverage surveys, secondary data analysis or anecdotal evidence). In such situations, they may choose to skip the assessment phase and convene brainstorming sessions to explore opportunities to intervene and reduce MOV. This model of implementation was recently piloted successfully in Cambodia in 2017. Similarly, although the use of electronic tablets for data collection shortens the interval between data collection and availability of results, their use may necessitate specific external technical assistance for electronic data management. As a compromise, countries may prefer to collect data on paper forms and delay the brainstorming session by a few days, to enable subsequent data entry and analysis.

As global partners and countries continue to work towards the GVAP goals, we hope that more countries with varying levels of immunization program performance will explore whether assessing MOV could be beneficial for diagnosing bottlenecks and improving their programs. We have shown that many unvaccinated children do make contact with health facilities and yet do not receive the recommended vaccine doses. In both countries, the findings of the assessments point to two major barriers to full vaccination of eligible children—a lack of coordination between vaccination and curative health services and incomplete vaccination during vaccination visits. National immunization programs should explore tailored efforts to improve health worker practices and to increase vaccine delivery by making better use of existing health service contacts to improve childhood vaccination.

## Supporting information

S1 File Missed opportunities for vaccination assessment exit interview survey (French): Chad, 2015. (PDF)

S2 File Missed opportunities for vaccination assessment health workers survey (French): Chad, 2015. (PDF)

S3 File Missed opportunities for vaccination assessment exit interview survey (English): Malawi, 2015. (PDF)

S4 File Missed opportunities for vaccination assessment health worker survey (English): Malawi, 2015. (PDF)
