# Supplementary material for: Can vaccination coverage be improved by reducing missed opportunities for vaccination? Findings from assessments in Chad and Malawi using the new WHO methodology
Source: PLoS One. 2019 Jan 24;14(1):e0210648. doi: 10.1371/journal.pone.0210648 (PMC6345496; doi:10.1371/journal.pone.0210648)
Supplement: S1 File — (PDF) [file pone.0210648.s001.pdf]

ANNEXE 4 : ENQUÊTE DE SORTIE DES ÉTABLISSEMENTS DE SANTÉ

Bonjour. Je m'appelle _______________________________ et je réalise une évaluation du programme de vaccination des enfants dans votre région. Notre objectif est de permettre l'amélioration générale du programme de vaccination. J'aimerais vous demander de nous aider en répondant aux questions de cette enquête. Je sais que vous êtes très occupé, cela ne prendra que quelques minutes.

Votre participation est volontaire et anonyme.
Souhaiteriez-vous répondre à mes questions ? Nous vous en remercions chaleureusement.*

Numéro de série du questionnaire pour la formation sanitaire: ______/___

A. Type de service
1. Service public/gouvernemental ☐
2. Privé ☐
4. Autre ☐ Précisez : ______________________________

Date de l'enquete
Jour /__/__/ Mois /__/__/ Année /__/__/

B. Type d'établissement de santé*
1. Hôpital ☐
2. Clinique ☐
3. Centre de santé ☐

C. Filtre
L'enfant qui accompagne l'adulte semble avoir moins de 24 mois
1. Oui ➡ POURSUIVEZ
2. Non ➡ REMERCIEZ LA PERSONNE ET MENTIONNEZ LA RENCONTRE CI-DESSOUS

SITUATION GÉOGRAPHIQUE

État/Région ______________________________________
District ______________________________________
Nom de l'établissement ______________________________________
Date de l'entretien ______________________________________

Nom
Enquêteur __________________________
Superviseur __________________________

Heure de début	*matin*	*après-midi*
Heure		
Minutes		

DONNÉES SUR L'ENFANT

(SI L'ADULTE ACCOMPAGNE PLUS D'UN ENFANT, SEUL LE PLUS JEUNE SERA PRIS EN COMPTE DANS L'ÉVALUATION, DANS LE CAS DE JUMEAUX, CHOISIR CELUI QUI EST NÉ LE DERNIER)

1. Date de naissance — Jour /__/__/ Mois /__/__/ Année /__/__/
   1.1 Âge de l'enfant — 99 NSP (Ne sait pas) / PR (Pas de réponse)
   1. Années ________
   2. Mois ________
   3. Semaines ________

2. Sexe
Masculin ☐ Féminin ☐

3. Pourquoi avoir amené l'enfant à cet établissement de santé aujourd'hui ? (NE PAS LIRE LES PROPOSITIONS)
1. Pour une consultation médicale (enfant malade) ☐
2. Pour une vaccination ☐
3. Bilan de santé/de croissance ou visite d'un enfant en bonne santé ☐
4. Pour accompagner la personne qui le soigne ou son frère/sa sœur à l'établissement de santé ☐
5. Hospitalisation ☐
6. Autre ☐ Précisez : ____________________

DONNÉES SOCIODÉMOGRAPHIQUES SUR LA PERSONNE QUI ACCOMPAGNE L'ENFANT, SON PARENT/TUTEUR

4. Âge (ans) : ________

5. Sexe 1. Masculin ☐ 2. Féminin ☐

* Comprend d'autres types d'établissement de soins comme les cabinets médicaux, les dispensaires, les cliniques temporaires et les centres de vaccination.

6. Quel lien avez-vous avec l'enfant ?

1. Mère ☐
2. Père ☐
3. Grand-parent ☐
4. Oncle/tante ☐
5. Frère/sœur ☐
6. Autre ☐ Précisez : ____________________

7. Situation familiale :

1. Célibataire ☐
2. Marié(e) ☐
3. En concubinage ☐
4. Séparé(e)/divorcé(e) ☐
5. Veuf/veuve ☐

8. Scolarisation

1. Aucune mais sait lire et écrire ☐
2. Aucune, ne sait ni lire ni écrire ☐
3. Primaire ou moins ☐
4. Secondaire, non achevé ☐
5. Secondaire achevé ☐
6. Au-delà du secondaire ☐

9. Métier

1. Ménagère (s'occupe des tâches domestiques) ☐
2. Employé ou ouvrier ☐
3. Travailleur indépendant ☐
4. Patron ou employeur ☐
5. Autre ☐ Précisez : ____________________

10. Combien de personnes composent votre ménage en comptant votre enfant ?

1. 2-5 ☐
2. 6 ou plus ☐
99 NSP/PR ☐

11. Quartier/Village/ville de résidence ______________________

12.Ce centre de santé se trouve-t-il dans le quartier/ village/la ville où vous résidez

1. Oui ☐ ➡ PASSEZ À LA QUESTION 14
2. Non ☐ ➡ POURSUIVEZ AVEC LA QUESTION 13
3. NSP ☐ ➡ PASSEZ À LA QUESTION 14

13. Pourquoi être venu dans cet établissement ?

1. Absence de services de santé dans mon village/ville de résidence ☐
2. Il y a des services de santé dans la commune où j'habite mais ils traitent mal les patients ☐
3. Cet établissement est sur mon trajet vers mon lieu de travail ☐
4. Cet établissement est dans le/la même village/ville que la garderie ou l'école de l'enfant ☐
5. Cet établissement propose de nombreux services de santé ☐
6. C'est ici que j'ai toujours amené cet enfant ☐
7. Autre ☐ Précisez : ____________________

14. Depuis combien de temps vivez-vous dans ce village/cette ville ?

1. Toujours ☐
2. _______ ans ☐
3. _______ mois ☐
4. _______ semaines ☐

5. _______ jours ☐

15. Quel moyen de transport utilisez-vous habituellement pour venir dans cet établissement ?

1. Marche ☐
2. Vélo ☐
3. Moto ☐
4. Voiture ☐
5. Bus ☐
6. Autre ☐ Précisez : ____________________

16. Combien de temps mettez-vous pour venir ici ? __________ Heures _________ Minutes

17. Le mois dernier, avez-vous entendu ou vu des messages sur la vaccination ?

1. Oui ☐ ➡ POURSUIVEZ AVEC LA QUESTION 18
2. Non ☐ ➡ PASSEZ À LA QUESTION 21

18. Où/Comment avez-vous entendu ou vu ces messages ?

1. Radio ☐
2. Télévision ☐
3. Journaux ☐
4. Établissement de santé ☐
5. Message téléphonique ☐
6. Facebook ou internet ☐
7. École des enfants ☐
8. Église ☐
9. Au cours d'une visite à domicile des agents de santé ☐
10. Autre ☐ Précisez : ____________________

19. Quel était le message ? ____

20. En quoi cette information vous a été utile ? **(NE PAS LIRE LES PROPOSITIONS)**

1. Savoir où faire vacciner l'enfant ☐
2. Avoir plus d'informations sur les vaccins ☐
3. Décider de faire vacciner l'enfant ☐
4. Aucune utilité ☐
5. Autre ☐ Précisez : ____________________

21. Quelle est votre source d'information sur la vaccination ? **(NE PAS LIRE LES PROPOSITIONS)**
PLUSIEURS RÉPONSES SONT POSSIBLES

1. Ne cherche pas d'informations ☐ ➡ PASSEZ À LA QUESTION 23
2. Radio ☐
3. Télévision ☐
4. Journaux ☐
5. Internet ☐
6. Carte de vaccination, carnet ou passeport de santé ☐
7. Autres parents/membres de la famille ☐
8. École des enfants ☐
9. Église, mosquée ou autre lieu de prière ☐
10. Établissements de santé ☐
11. Autre ☐ Précisez : ____________________

22. Comment utilisez-vous les informations trouvées sur la vaccination ?

1. Savoir où faire vacciner l'enfant ☐
2. Avoir plus d'informations que celles données par les services de santé ☐
3. Décider de faire vacciner l'enfant ☐
4. Aucune utilité ☐
5. Autre ☐ Précisez : ____________________

24. Avez-vous déjà fait vacciner votre enfant ?

1. Oui ☐
2. Non ☐

24.1 Avez-vous déjà demandé une vaccination pour cet enfant qui vous a été refusée ?

1. Oui ☐
2. Non ☐ ➡ PASSEZ À LA QUESTION 25

24.2 Si oui, pourquoi n'ont-ils pas vacciné l'enfant ?

1. Le médecin ou l'infirmier a dit que cela n'était pas possible car l'enfant était malade ☐
2. Ils n'avaient pas de vaccins ou ils n'avaient pas de seringues ou d'autre matériel nécessaire à la vaccination ☐
3. Ce n'était pas une journée de vaccination ☐
4. La zone de vaccination était fermée ☐
5. La personne responsable de la vaccination était absente ☐
6. Il y avait beaucoup d'attente ☐
7. Le personnel ne nous a pas bien traité ☐
8. Nous n'avions pas le passeport/carnet de vaccination ☐
9. Les horaires de vaccination étaient limités ☐
10. Autre ☐ Précisez : ______________________

25. Dans votre foyer, qui prend la décision de faire vacciner les enfants (PLUSIEURS RÉPONSES POSSIBLES)

1. Père ☐
2. Mère ☐
3. Autres parents ☐
4. Accord entre le père et la mère ☐
5. Autre ☐ Précisez : ______________________

UTILISATION DE LA CARTE DE VACCINATION/PASSEPORT DE SANTÉ ET INFORMATIONS SUR LES VACCINS ADMINISTRÉS

26. Votre enfant a-t-il un carnet de vaccination/passeport de santé ?

1. Oui et je l'ai apporté ☐ ➡ QUESTION 27
2. Oui, mais je ne l'ai pas avec moi ☐ ➡ QUESTION 26.1
3. Non ☐ ➡ QUESTION 26.1

26.1 À toutes les personnes interrogées, tout particulièrement si la personne n'a pas apporté le carnet de vaccination ou le passeport de santé aujourd'hui, demandez leur de fournir les informations demandées dans le Tableau ci-dessous
Précisez-leur que ces informations seront uniquement utilisées pour faire correspondre les notes dans le dossier de l'établissement de santé.

26.1.1	Identifiant sur la tablette	__________________
26.1.2	Prénom de l'enfant	__________________
26.1.3	Nom de famille de l'enfant	__________________
26.1.4	Adresse	__________________
26.1.5	Sexe	__________________
25.1.6	Date de naissance	__________________

➡ QUESTION 28

26.1 À la fin de l'entretien, utilisez les informations ci-dessus pour remplir le Tableau 27
26.3 N'oubliez pas de photographier la page

27. Demandez et vérifiez le carte de vaccination/passeport de santé de l'enfant ou son document temporaire de vaccination pour remplir le tableau suivant. ➡ PUIS PASSEZ À LA QUESTION 29

27.1 N'oubliez pas de photographier toutes les pages remplies du carnet/passeport de santé

Tableau 27

Quels vaccins a reçu l'enfant ?	A.	B.	C.

	Dates de toutes les doses administrées			Rappel	Administré aujourd'hui ?	
	JOUR	MOIS	ANNÉE		OUI	NON
1. BCG	/__/__/	/__/__/	/__/__/	À la naissance	☐	☐
2. VPO0	/__/__/	/__/__/	/__/__/	0 - 14 jours	☐	☐
3. VPO1	/__/__/	/__/__/	/__/__/	À 8 semaines	☐	☐
4. Rota1	/__/__/	/__/__/	/__/__/	À 8 semaines	☐	☐
5. DTC1/Penta1	/__/__/	/__/__/	/__/__/	À 8 semaines	☐	☐
6. PCV1	/__/__/	/__/__/	/__/__/	À 8 semaines	☐	☐
7. VPO2	/__/__/	/__/__/	/__/__/	À 12 semaines	☐	☐
8. Rota2	/__/__/	/__/__/	/__/__/	À 12 semaines	☐	☐
9. DTC2/Penta2	/__/__/	/__/__/	/__/__/	À 12 semaines	☐	☐
10. PCV2	/__/__/	/__/__/	/__/__/	À 12 semaines	☐	☐
11. VPO3	/__/__/	/__/__/	/__/__/	À 16 semaines	☐	☐
12. DTC3/Penta3	/__/__/	/__/__/	/__/__/	À 16 semaines	☐	☐
13. PCV3	/__/__/	/__/__/	/__/__/	À 16 semaines	☐	☐
14. Rota 3	/__/__/	/__/__/	/__/__/	À 16 semaines	☐	☐
15. RR1	/__/__/	/__/__/	/__/__/	À 9 - 11 mois	☐	☐
16. VAA	/__/__/	/__/__/	/__/__/	À 9 - 11 mois	☐	☐
17. RR2	/__/__/	/__/__/	/__/__/	À 15 - 18 mois	☐	☐

27.2 Où avez-vous trouvé les informations du Tableau 27 ?
1. Carte de vaccination/passeport de santé ☐
2. Document temporaire de vaccination ☐
3. Registre de l'établissement de santé ☐

28. Pourriez-vous nous dire pourquoi vous n'avez pas le carnet de vaccination/passeport de santé avec vous aujourd'hui ?
1. Il est à la maternelle/à la garderie ☐
2. Je l'ai laissé à la maison (je n'ai pas pensé à l'apporter) ☐
3. Je l'ai laissé à la maison (je ne savais pas que c'était important de l'apporter) ☐
4. Je l'ai perdu ☐
5. Le carnet/passeport de santé a été endommagé ☐
6. On ne m'en a jamais donné ➡ PASSEZ À LA QUESTION 31 ☐
7. Je n'étais pas venu pour une vaccination ☐
8. Autre ☐ Précisez : ______________________

29. Avez-vous déjà perdu le carnet de vaccination/passeport de santé de cet enfant ?
1. Oui ☐
2. Non ☐ ➡ PASSEZ À LA QUESTION 31

30. Avez-vous rencontré des difficultés pour le faire remplacer ?
1. Oui ☐
2. Non ☐

31. Pouvez-vous me dire à quoi sert le carnet de vaccination/passeport de santé ?
1. Pour savoir quels vaccins l'enfant a reçu et quels vaccins doivent être administrés ☐
2. Autre ☐ Précisez : ______________________
3. NSP/PR ☐

32. Aujourd'hui, au cours de votre visite dans l'établissement de santé, le personnel/l'équipe vous a-t-il/elle demandé le carnet de vaccination/passeport de santé de l'enfant ?
1. Oui ☐
2. Non ☐
3. Non, mais on m'a demandé si l'enfant était vacciné ☐

DONNÉES DE VACCINATION

33. Votre enfant a-t-il été vacciné au cours de cette visite dans l'établissement de santé ?

1. Oui ☐ ➡ PASSEZ À LA QUESTION 35
2. Non ☐

34. Pourquoi n'ont-ils pas vacciné l'enfant ? (Veuillez NE PAS lire les propositions)

ÉCOUTEZ D'ABORD LES RAISONS DONNÉES PAR LA PERSONNE QUI ACCOMPAGNE L'ENFANT PUIS ESSAYEZ DE CHOISIR LES BONNES PROPOSITIONS DANS LES BLOCS A, B OU C CI-DESSOUS

A. RAISONS LIEÉS AUX AGENTS DE SANTÉ

1. Le médecin/l'infirmier a dit que l'enfant était déjà vacciné, avait déjà reçu toutes les doses ou n'avait pas besoin d'être vacciné pour le moment ☐
2. Les agents de santé ne me l'ont pas demandé ☐
3. Le médecin/l'infirmier a dit que l'enfant ne pouvait pas être vacciné car il/elle était malade ☐

Types de maladies ou de traitements reçus aujourd'hui (cochez toutes les réponses correspondantes)

1. Rhume et/ou toux ☐
2. Diarrhée ☐
3. Parasitose intestinale ☐
4. Pneumonie ☐
5. Malnutrition et/ou anémie ☐
6. Légère fièvre ☐
7. Faible poids à la naissance ☐
8. Déshydratation ☐
9. L'enfant prend des médicaments : notez leur nom générique ________________ ☐
10. Infection des voies urinaires ☐
11. Autre maladie ☐ Précisez : ____________________

Enquêteur : SI LES RAISONS DONNÉES PAR LA PERSONNE INTERROGÉE CORRESPONDENT AU BLOC A CI-DESSUS,
➡ PASSEZ À LA QUESTION 45

B. RAISONS LIÉES A LA PERSONNE QUI ACCOMPAGNE L'ENFANT

1. La dernière fois que l'enfant a été vacciné, il/elle est tombé(e) malade ou a fait une réaction. ☐
2. Ma religion ne l'autorise pas. ☐
3. Expériences négatives d'un membre de la famille ou d'une connaissance. ☐
4. Je ne fais pas confiance aux vaccins de l'établissement de santé ☐
5. Je ne fais pas confiance au personnel des établissements de santé ☐
6. J'ai oublié ☐
7. Les vaccins peuvent entraîner des maladies ou une gêne ☐
8. Je n'ai pas le temps ☐
9. Les vaccins ne sont pas nécessaires ou je ne crois pas aux effets des vaccins ☐
10. L'enfant a reçu une vaccination complète ☐
11. La vaccination n'était pas le but de cette visite ☐
12. Autre ☐ Précisez : ____________________

Enquêteur : SI LES RAISONS DONNÉES PAR LA PERSONNE INTERROGÉE CORRESPONDENT AU BLOC B CI-DESSUS,
➡ PASSEZ À LA QUESTION 45

C. RAISONS LIÉES À LA LOGISTIQUE ET À L'ORGANISATION DE L'ÉTABLISSEMENT DE SANTÉ

1. Il n'y avait pas de vaccins ☐
2. Il n'y avait pas de seringues ou d'autre matériel nécessaire à la vaccination ☐
3. Ce n'est pas une journée de vaccination dans cet établissement de santé ☐
4. La zone de vaccination était fermée ☐
5. La personne responsable des vaccinations n'était pas là ☐
6. Il y avait beaucoup d'attente ☐
7. Le personnel nous a mal traité ☐
8. Les horaires de vaccination sont limités ☐
9. Autre ☐ Précisez : ____________________

Enquêteur : SI LES RAISONS DONNÉES PAR LA PERSONNE INTERROGÉE CORRESPONDENT AU BLOC C CI-DESSUS,
➡ **PASSEZ À LA QUESTION 45**

35. Combien de temps avez-vous attendu aujourd'hui pour que votre enfant soit vacciné ?
_________ Heures _____ Minutes

QUALITÉ DU SERVICE DE VACCINATION

36. Aujourd'hui, vous ont-ils dit quels vaccins étaient administrés à l'enfant ?
1. Oui ☐
2. Non ☐
3. PR ☐

37. Aujourd'hui, vous ont-ils donné la date du prochain rendez-vous de vaccination et l'ont-ils écrite sur le carnet de vaccination/passeport de santé ?
1. Oui ☐
2. Non ☐

39. Aujourd'hui, vous a-t-on informé des possibles « réactions » pouvant apparaître suite à une vaccination ?
1. Oui ☐
2. Non ☐ ➡ **PASSEZ À LA QUESTION 41**

40. Si oui, que vous ont-ils dit ? ____
____

41. Aujourd'hui, vous a-t-on informé sur ce que vous devez faire au cas où l'enfant « réagirait » aux vaccins ?
1. Oui ☐
2. Non ☐

42. Êtes-vous satisfait du service fourni aujourd'hui ?
1. Oui ☐
2. Non ☐ ➡ **PASSEZ À LA QUESTION 44**

43. Pourquoi ?
1. Attention immédiate ☐ ➡ **PASSEZ À LA QUESTION 45**
2. Le personnel nous a traité aimablement ☐ ➡ **PASSEZ À LA QUESTION 45**
3. Le service était gratuit ☐ ➡ **PASSEZ À LA QUESTION 45**
4. Autre ☐ Précisez : __________ ➡ **PASSEZ À LA QUESTION 45**

44. Pourquoi n'avez-vous pas été satisfait ?
1. Attente trop longue ☐
2. Le personnel était peu courtois ☐
3. Le langage utilisé par les agents de santé n'était pas clair ☐
4. Ils n'ont pas expliqué quels vaccins ils donnaient à l'enfant ☐
5. Autre ☐ Précisez : __________ ☐

45. Vous a-t-on déjà demandé de payer les vaccins administrés à un enfant ?
1. Oui ☐
2. Non ☐ ➡ **PASSEZ À LA QUESTION 47**

46. L'établissement de santé qui m'a demandé de payer était :
1. Public ☐
2. Privé ☐
3. NSP ☐

RAISONS DE LA VACCINATION

47. Pourriez-vous me dire l'objectif de la vaccination ? (cochez toutes les réponses correspondantes)

Veuillez NE PAS lire les propositions

1. Éviter les maladies ☐
2. Permettre aux enfants de grandir en bonne santé ☐
3. Guérir les maladies ☐
4. Cela n'apporte rien de bien ☐
5. Je ne sais pas trop à quoi cela sert ☐

48. De quelles maladies nous protègent les vaccins ? (cochez toutes les réponses correspondantes)

Veuillez NE PAS lire les propositions

1. Tuberculose ☐
2. Hépatite ☐
3. Poliomyélite ou polio ☐
4. Diphtérie ☐
5. Coqueluche ☐
6. Tétanos ☐
7. Pneumonie ☐
8. Méningite ☐
9. Diarrhée ☐
10. Grippe ☐
11. Rougeole ☐
12. Rubéole ☐
13. Oreillons ☐
14. Fièvre jaune ☐
15. Cancer ☐ Précisez : ___________
16. Autre ☐ Précisez : ___________
17. Aucune ☐
18. NSP ☐

49. Pensez-vous que votre enfant peut attraper des maladies si vous ne le vaccinez pas ?

1. Oui ☐
2. Non ☐
3. NSP ☐

50. Que suggérez-vous pour améliorer les services de vaccination ?

1. Il devrait y avoir plus de personnel de vaccination ☐
2. Il devrait y avoir moins d'attente ☐
3. Les horaires et jours de vaccination ne devraient pas être limités ☐
4. Les carnets de vaccination ne devraient pas être distribués ☐
5. Le traitement du public et des enfants à vacciner devrait être plus agréable ☐
6. Le centre de santé devrait toujours avoir des vaccins ☐
7. Ils devraient fournir des informations sur les vaccins administrés, sur les maladies qu'ils évitent et sur les réactions qu'ils entraînent. ☐
8. Autre ☐ Précisez : ____________________ ☐
9. Rien ☐
10. NSP ☐
11. PR ☐

Enquêteur : Remerciez la personne interrogée et notez à quelle heure l'entretien s'est terminé.
Lisez la déclaration suivante : « N'oubliez pas que la vaccination est un droit pour tous. Réclamez ce droit et pensez à apporter le carnet de vaccination de votre enfant à l'établissement de santé chaque fois que vous vous y rendez, pour quelque raison que ce soit. »

Heure de fin : Heure : |__|__| Minutes : |__|__|

Commentaires de l'enquêteur : ____

____

____

Superviseur : Veuillez vérifier l'exactitude et l'exhaustivité du formulaire complété

1. Le formulaire a été rempli de manière précise et totale (l'enchaînement des questions a été respecté) ☐
2. Erreurs ou incohérences sur le formulaire ☐

Commentaires du superviseur : ____

____

____

Nom et prénom du superviseur : ____

Signature du superviseur : ____
