# Supplementary material for: Can vaccination coverage be improved by reducing missed opportunities for vaccination? Findings from assessments in Chad and Malawi using the new WHO methodology
Source: PLoS One. 2019 Jan 24;14(1):e0210648. doi: 10.1371/journal.pone.0210648 (PMC6345496; doi:10.1371/journal.pone.0210648)
Supplement: S2 File — (PDF) [file pone.0210648.s002.pdf]

ANNEXE 5 : ENQUÊTE AUPRÈS DES AGENTS DE SANTÉ

Le Ministère de la Santé, en collaboration avec le Bureau régional de l'Organisation mondiale de la santé pour l'Afrique, souhaite renforcer les compétences techniques de tous les agents de santé qui fournissent des services de vaccination. Ce questionnaire a été élaboré afin d'identifier les thèmesde formation à venir sur la vaccination. Nous vous remercions chaleureusement de votre collaboration. Ce questionnaire est anonyme et votre participation est volontaire. Si vous décidez de participer, veuillez utiliser un crayon ou un stylo pour noter les réponses qui, selon vous, correspondent le mieux à la question ou au problème présenté. Les réponses ne seront en aucun cas utilisées pour évaluer vos capacités professionnelles. Lisez attentivement chaque partie de ce questionnaire et veillez à répondre à toutes les questions.

Numéro de série du questionnaire pour la formation sanitaire: _______/____

Date de l'enquete

Jour /__/__/ Mois /__/__/ Année /__/__/

A. Type de service

1. Service public/gouvernemental ☐
2. Privé ☐
4. Autre ☐ Précisez : ______________________________

B. Type d'établissement de santé

1. Hôpital ☐
2. Clinique ☐
3. Centre de santé ☐

I. INFORMATIONS GÉNÉRALES

Veuillez cocher la bonne réponse

1. Sexe 1. Masculin ☐ 2. Féminin ☐

2. Âge |___|___| ans

3. Formation professionnelle
   - 3.1 Médecin généraliste ☐
   - 3.2 Spécialiste (comprend un diplôme de master) ☐
   - 3.3 Infirmier/sage-femme/Attaché de santé ☐
   - 3.4 Aide soignant/AIS/Accoucheuses auxilaires ☐
   - 3.5 Agent de santé communautaire ☐
   - 3.6 Autre ☐ Précisez : ____________________

4. Domaine dans lequel vous travaillez
   - 4.1 Consultations externes, hospitalisations, urgences ☐
   - 4.2 Médecine préventive, épidémiologie ou vaccination ☐

5. Ancienneté |___|___| ans |___|___| mois

6. Avez-vous suivi une formation ou participé à des cours sur la vaccination ou les maladies évitables par la vaccination ?
   1. Oui ☐
   2. Non ☐ ➡ PASSEZ À LA QUESTION 8

7. Si oui, à quand remonte votre dernière formation ?
   1. - d'1 an ☐
   2. entre 1 et 2 ans ☐
   3. entre 2 et 3 ans ☐
   4. 4 ans et + ☐

8. Des séances d'information ou de formation cliniques sont-elles organisées dans votre hôpital ou votre centre de santé ?
   1. Oui ☐
   2. Non ☐ ➡ PASSEZ À LA QUESTION 10

9. Si oui, le thème de la vaccination ou des MEV a-t-il été abordé ces 12 derniers mois ?
   1. Oui ☐
   2. Non ☐

II. CONNAISSANCES SUR LA VACCINATION

POUR LES QUESTIONS 10 À 13, VEUILLEZ COCHER TOUTES LES BONNES RÉPONSES

10. Les vaccins pouvant être administrés à des personnes en bonne santé sont :
   1. BCG ☐
   2. Rougeole ☐
   4. DTP/Pentavalent ☐

11. Les maladies évitées grâce aux vaccins pentavalents :
   1. Hépatite B, coqueluche, tétanos, pneumonie et méningite à Haemophilus influenzae de type b ☐
   2. Diphtérie, coqueluche, tétanos, hépatite B et grippe ☐
   3. Poliomyélite, diphtérie, coqueluche, tétanos, pneumonie et méningite à pneumocoques ☐
   4. Diphtérie, coqueluche, tétanos, poliomyélite, pneumonie et méningite à Haemophilus influenza de type b ☐
   5. Aucune des propositions ci-dessus ☐

12. Les contre-indications à la vaccination contre le poliovirus comprennent :
   1. Allaitement ☐
   2. Température axillaire ou rectale de 37,5^{o} C ☐
   3. Malnutrition légère ☐
   4. Diarrhée légère ☐
   5. Aucune des propositions ci-dessus ☐

13. Âge recommandé pour l'administration des vaccins suivants. Inscrivez dans la colonne vierge du premier tableau le nombre correspondant à la bonne réponse dans le 2e tableau

**1**	DTP/Pentavalent	
**2**	Fièvre jaune	
**3**	BCG	
**4**	VPO	
**5**	1ère dose RR	

**1**	À la naissance et à 8, 12 et 16 semaines
**2**	8, 12, 16 semaines
**3**	9 mois
**4**	12 mois
**5**	À la naissance

POUR LES QUESTIONS 15 À 17, VEUILLEZ COCHER UNE SEULE BONNE RÉPONSE

14. Vaccins utilisés pour éviter la pneumonie chez l'enfant de moins de 2 ans. Cochez une seule réponse :
   1. Grippe, hépatite B et vaccin pneumococcique conjugué ☐
   2. BCG, pentavalent et hépatite B ☐

3. Rotavirus, pentavalent et vaccin pneumococcique conjugué ☐
4. Pentavalent, grippe et vaccin pneumococcique conjugué ☐
5. Aucune des propositions ci-dessus ☐

15. Les MEV en cours d'éradication ou d'élimination sont :
1. Varicelle ☐
2. Coqueluche ☐
3. Poliomyélite ☐
4. Rougeole, rubéole et syndrome de rubéole congénitale ☐
5. Seules les propositions 3 et 4 sont bonnes ☐

16. Contre-indications à tous les vaccins :
1. Réaction locale à une dose précédente ☐
2. Légère fièvre ☐
3. Troubles apparus suite à un traitement médical ☐
4. Pneumonie ou autres maladies graves ☐
5. Aucune des propositions ci-dessus ☐

17. Pensez-vous que dans certains cas une personne vaccinée contre une maladie pourrait contracter cette maladie plusieurs années plus tard ?
1. Oui ☐
2. Non ☐
Précisez votre réponse : ____
____

19. Pour la liste de vaccins suivante, cochez la colonne AT si le vaccin est atténué ou cochez la colonne IN si le vaccin est inactivé, recombinant ou fractionné.

N°	Vaccins				AT	IN
**1**	BCG					
**2**	DTP					
**3**	Rougeole					
**4**	Rubéole					
**5**	VPO					
**6**	Haemophilus influenzae de type b (Hib)					
**7**	Hépatite					

III. COMPORTEMENT

POUR LES QUESTIONS 20 À 23, VEUILLEZ COCHER UNE SEULE BONNE RÉPONSE.

20. Au quotidien, qui s'occupe de l'état de vaccination des enfants, vérifie les carnets de vaccination /passeports de santé et s'assure que les enfants sont à jour par rapport au calendrier de vaccination national ?
1. Les parents de l'enfant ☐
2. L'infirmier responsable de la vaccination ☐
3. Les médecins des consultations externes, hospitalisations et urgences ☐
4. Toutes les personnes ci-dessus ☐
5. Seules les propositions 1 et 2 sont bonnes ☐

21. Dans laquelle des situations suivantes devriez-vous demander des informations sur les doses reçues par les enfants et celles qui manquent suivant leur âge ?
1. Visite de routine de l'enfant ☐

2. Consultation pour n'importe quelle maladie ☐
3. Lorsqu'un enfant accompagne la personne qui le soigne au cours d'un bilan de santé prénatal ☐
4. Lorsqu'un enfant accompagne la personne qui le soigne lors d'une visite à l'établissement de santé quelle que soit la raison ☐
5. Toutes les propositions ci-dessus ☐

22. Pourquoi pensez-vous que certains enfants ne sont pas à jour dans leurs vaccinations ?
1. Opinion négative des parents vis-à-vis de la vaccination ☐
2. Horaires de vaccination incompatibles avec les vies très chargées des parents ☐
3. Les médecins, infirmiers et agents de santé ne se renseignent pas sur les calendriers de vaccination des enfants ☐
4. Les médecins, infirmiers et agents de santé ne vérifient pas les carnets de vaccination /passeports de santé des enfants ☐
5. Fausses contre-indications à la vaccination par les agents de santé ☐
6. Éloignement par rapport au lieu de vaccination ☐
7. Toutes les propositions ci-dessus ☐

23. Pensez-vous que les vaccins administrés dans les établissements privés confessionnels sont différents en termes de qualité de ceux fournis par le Ministère de la Santé ?
1. Oui ☐
2. Non ☐
3. Ne sait pas ☐

24. Veuillez préciser votre réponse : ____
____

POUR LES QUESTIONS 25 À 28, VEUILLEZ INDIQUER SI VOUS ÊTES D'ACCORD OU NON AVEC LES DÉCLARATIONS SUIVANTES :

25. Mes connaissances concernant les vaccins et la vaccination sont insuffisantes ou dépassées.
1. D'accord ☐
2. Pas d'accord ☐

26. Mes connaissances sur la vaccination et le Programme élargi de vaccination (PEV) sont suffisantes pour répondre aux besoins de l'institution.
1. D'accord ☐
2. Pas d'accord ☐

27. J'ai peur des effets secondaires des vaccins.
1. D'accord ☐
2. Pas d'accord ☐

28. Remplir les registres nominaux de vaccination (livres/carnets) retarde la vaccination systématique des enfants.
1. D'accord ☐
2. Pas d'accord ☐

IV. PRATIQUES

IV.I PRISE DE DÉCISION DANS LA PRATIQUE QUOTIDIENNE
CETTE PARTIE CONCERNE TOUS LES PROFESSIONNELS DE SANTÉ

POUR LES QUESTIONS 29 À 32, VEUILLEZ COCHER LA BONNE RÉPONSE

29. Nourrisson de 3 mois, de sexe féminin, a déjà reçu à la naissance, une dose de BCG. La mère vient pour un contrôle de la croissance et du développement de l'enfant. Quels vaccins allez-vous administrer à l'enfant ?
    1. Aucun ☐
    2. Seulement la Polio ☐
    3. Seulement le Pentavalent ☐
    4. Le vaccin contre la rougeole ☐
    5. Le Polio et le Pentavalent ☐

30. Nouveau-né de sexe masculin, pesant 3,2 kg, naissance normale par voie basse dans un hôpital. La mère est séronégative au VIH mais est porteuse du virus de l'hépatite B. Quels vaccins
    1. BCG ☐
    3. Pentavalent ☐
    4. Aucune des propositions ci-dessus ☐
    5. Seules les propositions 1 et 2 sont bonnes ☐

31. Nourrisson de 6 mois, de sexe féminin, a déjà reçu une dose de BCG, une dose contre l'hépatite B, deux doses de pentavalent à cellules entières et deux doses de vaccin contre la polio. Les dernières doses ont été administrées lorsque l'enfant avait 4 mois. Selon la mère, il y a un mois, l'enfant a eu de la fièvre et une crise et suit actuellement un traitement médical. Suivant les recommandations du PEV, quels vaccins allez-vous lui administrer ?
    1. Je ne la vaccinerais pas ☐
    2. Seulement le vaccin contre la polio et je l'orienterais vers un spécialiste ☐
    3. Seulement le vaccin contre l'hépatite B ☐
    4. Le vaccin contre la polio et le Pentavalent ☐
    5. Seulement le vaccin contre la rougeole ☐

32. Quels vaccins avez-vous déjà administré à un adulte ? (cochez la/les réponse(s) correspondante(s))
    1. VAT ☐
    2. VAR ☐
    3. Hépatite B ☐
    4. VAA ☐
    5. VPO ☐
    6. MenA ☐
    7. Aucune des propositions ci-dessus ☐

IV. PRATIQUES

IV.2 PRISE DE DÉCISION ET PRATIQUES DE VACCINATION

CETTE PARTIE CONCERNE UNIQUEMENT LES PROFESSIONNELS DE SANTÉ QUI ADMINISTRENT LES VACCINS

33. Dans quels cas fourniriez-vous des conseils concernant la marche à suivre si l'enfant est victime d'effets secondaires suite à une vaccination ?
    1. Uniquement si le vaccin peut entraîner une réaction grave ☐
    2. Seulement si le parent ou le tuteur demande des renseignements ☐
    3. Jamais car ces informations peuvent être contre-productives et les dissuader de participer au programme de vaccination ☐

4. Toujours, peu importe le vaccin utilisé et les réactions pouvant en découler ☐
5. La probabilité d'un effet secondaire lié à la vaccination est si faible que j'ai très rarement besoin de fournir ces informations ☐

34. Aujourd'hui, vous administrez à une enfant de 2 mois les premières doses de Pentavalent et de vaccin contre la polio. Après avoir dit aux parents quels vaccins elle allait recevoir, quelles autres informations et conseils devriez-vous leur donner ?
    1. L'enfant peut avoir un peu de fièvre, de diarrhée ou une gêne après la vaccination ☐
    2. Généralement, les symptômes ci-dessus ne nécessitent pas de traitement. Toutefois, en cas de fièvre, veillez à ce que l'enfant ne soit pas trop couvert et n'arrêtez jamais l'allaitement. ☐
    3. Les parents doivent retourner au centre de santé si ces symptômes persistent afin que l'enfant voit un médecin. ☐
    4. Toutes les propositions ci-dessus ☐
    5. Aucune des propositions ci-dessus ☐

35. Que devriez-vous faire si vous remarquez sur les registres de vaccination du centre de santé des enfants dont les vaccins ne sont pas à jour ?
    1. Établir une liste hebdomadaire des enfants dont le calendrier est incomplet ☐
    2. Contacter les parents ou tuteurs par téléphone, courriel ou tout autre moyen de communication pour leur rappeler de vacciner leurs enfants ☐
    3. Faire des visites à domicile pour encourager les famille à compléter les calendriers de vaccination de leur enfant et en profiter pour administrer les doses manquantes ☐
    4. Toutes les propositions ci-dessus ☐
    5. Aucune des propositions ci-dessus ☐

36. Que pourrait-il être fait pour suivre la vaccination des enfants suite à une hospitalisation ou à un traitement en externe pour une maladie chronique ?
    1. Coordination entre les zones cliniques et les services d'urgences et d'hospitalisation de l'hôpital afin qu'ils puissent vérifier le carnet de vaccination/passeport de santé de l'enfant ☐
    2. Envoyer les patients pour lesquels les médecins valident la vaccination au service de vaccination afin qu'ils soient vaccinés avant de quitter l'hôpital ☐
    3. À l'hôpital, un agent de santé du service de vaccination pourrait visiter le service d'hospitalisation pour vérifier les dossiers médicaux des enfants qui sortiront dans la journée, et ainsi identifier les enfants qui devront commencer ou compléter leur calendrier de vaccination ☐
    4. Toutes les propositions ci-dessus ☐
    5. Aucune des propositions ci-dessus ☐

37. À 8h, vous préparer un porte vaccin pour l'équipe du matin dans l'établissement de santé. Conformément aux recommandations relatives à la chaîne du froid pour les portes vaccin, vous placez deux flacons de 10 doses de vaccin contre la rougeole dans les portes vaccin. À 15h, une mère demande que sa fille âgée de 13 mois reçoive une dose de vaccin contre la rougeole. Après avoir vérifié le carnet de l'enfant, vous vous apercevez que le vaccin contre la rougeole ne lui a jamais été administré mais qu'elle a toutefois déjà reçu tous les vaccins destinés aux enfants de - d'1 an. De plus, l'enfant ne présente pas de contre-indications. Seules deux doses du premier flacon ont été administrées depuis 8h30, lorsque la première dose a été administrée. Lequel des deux flacons de vaccin allez-vous utiliser pour vacciner cette enfant ? ___

1. J'utiliserais le flacon ouvert pour éviter de gaspiller du vaccin ☐
2. Je demanderais à la mère de revenir le jour suivant car je ne peux pas ouvrir un nouveau flacon de vaccin et il n'y a pas d'autres enfants à vacciner ☐
3. J'ouvrirais le second flacon de vaccin contre la rougeole pour vacciner la petite fille ☐
4. Je recommanderais à la mère d'emmener son enfant à un autre centre de santé pour la faire vacciner ☐
5. Aucune des propositions ci-dessus ☐

38a. Quelles instructions donnez-vous habituellement aux personnes qui accompagnent les enfant la première fois que vous leur remettez un carte de vaccination ? (VEUILLEZ COCHER TOUTES LES RÉPONSES CORRESPONDANTES)

1. Faites bien attention à ce carte ☐
2. Apportez ce carte à chaque fois que vous venez dans l'établissement de santé ☐
3. Apportez ce carte uniquement lorsque vous venez pour une vaccination ☐
4. Aucune instruction n'est donnée ☐
5. Autres : Précisez : ____________________________ ☐

38b. Que faites-vous lorsqu'une personne accompagnant un enfant a oublié le carte de vaccination de santé chez lui :

1. Je ne vaccine pas l'enfant et demande à la mère de revenir la prochaine fois avec le carte ☐
2. Je remets une nouvelle carte et note la vaccination du jour ☐
3. Je remets une nouvelle carte et note toutes les vaccinations déjà effectuées inscrites sur le registre ☐
4. Je remets une carte temporaire et lui demande d'apporter l'ancien carte lors de sa prochaine visite ☐
5. Autres : Précisez : ____________________________ ☐

38c. Si une femme indique que la carte de l'enfant a été perdu ou abîmé, que faites-vous généralement ?

1. Je remets une nouvelle carte et note tous les vaccins à venir ☐
2. Je remets une nouvelle carte et retranscrits tous les vaccins déjà effectués inscrits sur le registre ☐
3. Je remets une nouvelle carte et demande à la femme de m'indiquer toutes les vaccinations précédentes afin que je puisse les noter ☐
4. Autres : Précisez : ____________________________ ☐

39. Ci-dessous, dans la liste des ressources et procédures concernant la chaîne du froid, cochez la déclaration exacte si elle s'applique aux réfrigérateurs, aux glacières ou aux deux

N^{o}	Ressources et procédures	Réfrigérateur	Glacières
1	Stockage entre 2 et 8° C		
2	Accumulateurs de froid		
3	À 15 cm du mur		
4	Feuille de température		

DANS LA PARTIE SUIVANTE, INDIQUEZ VOTRE ACCORD OU DÉSACCORD AVEC LES DÉCLARATIONS SUIVANTES EN COCHANT LA CASE CORRESPONDANTE :

40. Dans cet établissement de santé, le personnel réalisant des services de vaccination est suffisant

1. Oui ☐
2. Non ☐

41. Aujourd'hui, j'ai assez de flacons de tous les vaccins pour les patients faisant appel aux services de vaccination

1. Oui ☐
2. Non ☐

42. Aujourd'hui, j'ai toutes les seringues, registre de pointage, carte de vaccination/ et tout autre matériel dont j'ai besoin pour vacciner les patients qui font appel à un service de vaccination

1. Oui ☐
2. Non ☐

43. Lorsque le responsable de la vaccination est absent, un autre agent de santé est disponible pour le/la remplacer

1. Oui ☐
2. Non ☐

COMMENTAIRES SUPPLÉMENTAIRES :

____
____
____
____
____

Nous vous remercions de votre participation et vous souhaitons une bonne journée !
