# Supplementary material for: Can vaccination coverage be improved by reducing missed opportunities for vaccination? Findings from assessments in Chad and Malawi using the new WHO methodology
Source: PLoS One. 2019 Jan 24;14(1):e0210648. doi: 10.1371/journal.pone.0210648 (PMC6345496; doi:10.1371/journal.pone.0210648)
Supplement: S3 File — (PDF) [file pone.0210648.s003.pdf]

ANNEX 4: HEALTH FACILITIES EXIT SURVEY

Good morning/afternoon. My name is ________________________________ and I am working on a program evaluation of the vaccination of children in this locality. Our goal is to help improve the vaccination program in general. I would like to respectfully ask for your help in aswering the questions in this survey. I know you are busy, so we will try to interview you for only a few minutes. Your participation is voluntary and anonymous. Would you be willing to answer these questions? Thank you very much.*

Questionnaire Serial Number ☐☐☐ THIS IS PRE-ASSIGNED CENTRALLY

Date of interview
Day |__|__| Month |___|___| Year |___|___|

GEOGRAPHICAL LOCATION

State/Province/Region ___________________
District ___________________
Name of facility _______________________________________
Interviewer __________________________
Supervisor __________________________

Start time	***am***	***pm***
Hour		
Minutes		

A. Type of service

1. Public/Government service ☐
2. Private ☐
3. NGO ☐
4. Faith-based organization ☐
5. Other ☐ Specify: ______________________________

B. Health facility type

1. Hospital ☐
2. Clinic * ☐
3. Health center ☐

* Includes other types of primary care facilities such as doctors’ offices, dispensaries, limited-service clinics, and vaccination posts.

C. Filter

The child appears to be <24 months

1. Yes ☐ ➡ CONTINUE (RECORD RESPONSE IN TRACKING FORM PLEASE)
2. No ☐ ➡ **THANK THE PERSON AND RECORD THE ENCOUNTER (TRACKING FORM)**

DATA ON THE CHILD
(IN CASE OF MORE THAN ONE CHILD, CHOOSE THE YOUNGEST CHILD)

1. Date of birth Day |___|___| Month |___|___| Year |___|___|
☐ DK (Doesn't know) / NR (No response)

1.1 Age of child
1. _____ years
2. _____ months
3. _____ weeks

2. Sex — 1. Male ☐ 2. Female ☐

3. Why did you bring the child to this health care facility today? (DO NOT READ OUT THE CHOICES)
1. For medical consultation (child is sick)
2. For vaccination
3. Healthy child visit or growth/development check-up
4. Child is only accompanying (not for treatment, vaccination)
5. Hospitalization (child was admitted or is still on admission)
6. Other ☐ Specify: ____________________

DEMOGRAPHICS ON CHILD'S CAREGIVER, PARENT/GUARDIAN

4. Sex — 1. Male ☐ 2. Female ☐

5. What is your relationship to the child?
1. Mother ☐
2. Father ☐
3. Grandparent ☐
4. Uncle/aunt ☐
5. Brother/sister ☐
6. Other ☐ Specify: ____________________

6a. Can you read and write?
1. Yes ☐
2. No ☐

6b. Level of formal education
1. No formal education ☐
2. Incomplete primary ☐
3. Complete primary, ☐
2. Incomplete secondary ☐
3. Completed secondary school ☐
4. More than secondary ☐

7. Marital status:
1. Single ☐
2. Married ☐
3. Separated/divorced ☐
4. Widowed ☐

7b. What do you do for a living?
1. Housewife (work is housekeeping) ☐
2. Employee or laborer ☐
3. Farming ☐
4. Self-employed ☐
5. Boss or employer ☐
6. Other ☐ Specify: ____________________

8. Village/town of residence __________________________

9. Is this health center in the village/area where you live
1. Yes ☐ ➡ SKIP TO QUESTION 11
2. No ☐ ➡ CONTINUE WITH QUESTION 10

10. Why do you prefer to come to this health facility?

1. No health services in my village/town of residence ☐
2. There are health services in the municipality where I live, but their treatment of patients is not good ☐
3. This facility is on the way to my workplace ☐
4. This facility is in the same village/town as the child's day care or school ☐
5. Because this facility offers various health services ☐
6. I have always brought this child here ☐
7. Other ☐ Specify: ____________________

11. How long have you been living in this village/town?

1. Always ☐
2. _______ years
3. _______ months
4. _______ weeks
5. _______ days

12. By what means of transportation do you usually come to this facility?

1. Walk ☐
2. Bicycle ☐
3. Motorcycle ☐
4. Car ☐
5. Bus ☐
6. Ox carts ☐
7. Other ☐ Specify: ____________________

13. How long does it take you to get here? _____Hours _____Minutes

14. Have you heard or seen messages on vaccination in the last one month?

1. Yes ☐ ➡ CONTINUE WITH QUESTION 15
2. No ☐ ➡ SKIP TO QUESTION 17

15. Where/How did you hear or see the messages? (CHECK ALL THAT APPLY)

1. Radio ☐
2. Television ☐
3. Newspaper ☐
4. Health facility ☐
5. Telephone message ☐
6. Facebook or internet ☐
7. Children's school ☐
8. Place of worship ☐
9. During home visit by health workers/health outreaches ☐
10. Community meetings ☐
11. Other ☐ Specify: ____________________

16. What did the message say? ____

17. Where do you usually look for information on what vaccines your child needs? **(DO NOT READ OUT THE OPTIONS. CHECK ALL THAT APPLY)**

1. Does not seek information ☐ ➡ SKIP TO QUESTION 18
2. Radio ☐
3. Television ☐
4. Newspaper ☐
5. Internet ☐
6. Vaccination booklet, health passport or card ☐
7. Other parents/family members ☐
8. Children's school ☐
9. Church or mosque or other place of worship ☐
10. Health facilities ☐
11. During home visit by health workers/health outreaches ☐
12. Other ☐ Specify: ____________________

18. How would you assess your level of information on vaccines or information on the need for vaccination?

1. Fairly adequate ☐
2. Adequate ☐
3. Inadequate ☐

19. Has this child ever been vaccinated?

1. Yes ☐
2. No ☐

19.1 Have you ever requested vaccination service for this child and been refused?

1. Yes ☐
2. No ☐ ➡ SKIP TO QUESTION 20

19.2 If so, why didn't they vaccinate the child?

1. The doctor or nurse said it couldn't be done because the child was sick ☐
2. There were no vaccines, or there were no syringes or some other supply needed for vaccination ☐
3. It was not a vaccination day ☐
4. The vaccination area was closed ☐
5. The person in charge of vaccination was not there ☐
6. We didn't have the vaccination card/passport ☐
7. The hours for vaccination are limited ☐
8. Other ☐ Specify: ____________________

20. In your home, who makes the decision to vaccinate the children

1. Father ☐
2. Mother ☐
3. Other relatives ☐
4. Consensus of father and mother ☐
5. Other ☐ Specify: ____________________

USE OF VACCINATION CARD/HEALTH PASSPORT AND INFORMATION ON VACCINES ADMINISTERED

21. Does your child have a vaccination card/health passport?

1. Yes, and I have it with me ☐ ➡ GO TO QUESTION 22
2. Yes, but I do not have it with me ☐ ➡ GO TO QUESTION 21.1
3. No ☐ ➡ GO TO QUESTION 21.1

21.1 Whenever the vaccination card or health passport is not with the caregiver today, request to complete the information in Annex 7 (Health Facility Register Follow-Up Form). Assure them that this information will only be used to match the records in the health facility register.
At the end of all the interviews, use the information in Annex 7 to complete Table 22.
Remember to ALSO take a picture of the relevant pages/lines of the register. ➡ **GO TO 23**

22. Request and examine the child's vaccination card/health passport or temporary vaccination document to fill out the following table. ➡ THEN GO TO QUESTION 24

22.1 Remember to take pictures of all the completed pages on the health passport/card

Table 22

What vaccines has this child received?	A. Dates of all doses given DAY	MONTH	YEAR	B. Reminder	C. Vaccine given, BUT date illegible YES	NO
1. BCG	|___|___|	|___|___|	|___|___|	At birth	☐	☐
2. OPV0	|___|___|	|___|___|	|___|___|	0 - 14 days	☐	☐
3. OPV1	|___|___|	|___|___|	|___|___|	At 6 weeks	☐	☐
4. Rota1	|___|___|	|___|___|	|___|___|	At 6 weeks	☐	☐
5. Penta1/DPT1	|___|___|	|___|___|	|___|___|	At 6 weeks	☐	☐
6. PCV1	|___|___|	|___|___|	|___|___|	At 6 weeks	☐	☐
7. OPV2	|___|___|	|___|___|	|___|___|	At 10 weeks	☐	☐
8. Rota2	|___|___|	|___|___|	|___|___|	At 10 weeks	☐	☐
9. Penta2/DPT2	|___|___|	|___|___|	|___|___|	At 10 weeks	☐	☐
10. PCV2	|___|___|	|___|___|	|___|___|	At 10 weeks	☐	☐
11. OPV3	|___|___|	|___|___|	|___|___|	At 14 weeks	☐	☐
12. Penta3/DPT3	|___|___|	|___|___|	|___|___|	At 14 weeks	☐	☐
13. PCV3	|___|___|	|___|___|	|___|___|	At 14 weeks	☐	☐
14. IPV	|___|___|	|___|___|	|___|___|	At 14 weeks	☐	☐
15. Measles1	|___|___|	|___|___|	|___|___|	At 9 - 11 months	☐	☐
16. Yellow fever	|___|___|	|___|___|	|___|___|	At 9 - 11 months	☐	☐
17. Measles2	|___|___|	|___|___|	|___|___|	At 15 - 23 months	☐	☐

22.2 What was the source of the information in Table 22

1. Vaccination card/health passport ☐
2. Temporary vaccination document ☐
3. Health facility register ☐

23. Could you tell us why you do not have the vaccination card/health passport with you today?

1. It is at the nursery school/day care center ☐
2. I left it at home (because I forgot to bring it) ☐
3. I left it home (because I didn't know it was important to bing it along) ☐
4. I lost it ☐
5. The card/health passport has been damaged ☐
6. I have never been given one ➡ SKIP TO QUESTION 26 ☐
7. Because vaccination was not the reason for this visit ☐
8. Other ☐ Specify: ______________________

24. Have you ever lost a vaccination card/health passport for this child?

1. Yes ☐
2. No ☐ ➡ **SKIP TO QUESTION 26**

25. Did you encounter difficulty getting it replaced?

1. Yes ☐
2. No ☐

26. Could you tell me what purpose the vaccination card/health passport serves?

1. To know what vaccines the child has had and which ones are missing ☐
2. Other ☐ Specify: ____________________
3. Don't Know/No Response ☐

27. During today's visit, did the personnel/staff ask you for the child's vaccination card/health passport?

1. Yes ☐ ➡ **SKIP TO QUESTION 28**
2. No ☐ ➡ **SKIP TO QUESTION 27b**

27b. If No, did they ask for the vaccination status of the child?

1. Yes ☐
2. No ☐

TODAY'S VACCINATION

28. Was your child vaccinated here today?

1. Yes ☐ ➡ **SKIP TO QUESTION 35**
2. No ☐

---

29. Why didn't they vaccinate the child? (Please DO NOT read the options)

FIRST LISTEN TO THE REASONS GIVEN BY THE CAREGIVER AND THEN TRY TO CHOOSE THE RIGHT OPTION FROM BLOCK A, B OR C BELOW

A. REASONS RELATED TO THE HEALTH WORKERS

1. The doctor/nurse said that the child is already vaccinated, or has had the complete series, or is not due for a vaccine at this time ☐
2. The health workers did not ask me ☐
3. The doctor/nurse said that the child could not be vaccinated because he/she was sick and treated today ☐

Type of disease or treatment received today (check all that apply)

1. Cold and/or cough ☐
2. Diarrhea ☐
3. Intestinal parasitosis ☐
4. Pneumonia ☐
5. Malnutrition and/or anemia ☐
6. Mild fever ☐
7. Low birth weight ☐
8. Dehydration ☐
9. Child is taking medications: ☐ Write down generic name ______________
10. Urinary tract infection ☐
11. Other ☐ Specify: ____________________

Interviewer: IF THE REASONS CITED BY THE INTERVIEWEE REFER TO BLOCK A ABOVE, SKIP TO QUESTION 39 ➡

B. REASONS RELATED TO THE CAREGIVER

1. The last time the child was vaccinated, he/she got sick or had a reaction. ☐
2. My religion doesn't permit it. ☐
3. Negative experiences of a family member or acquaintances. ☐
4. I don't trust the health facility's vaccines ☐
5. I don't trust personnel of the health facilities ☐
6. I forgot ☐
7. Vaccines can cause some disease or discomfort ☐
8. I don't have time ☐
9. Vaccines are not necessary, or I don't believe in vaccines ☐
10. The child has completed the series ☐
11. Vaccination was not the purpose of this visit ☐
12. Other ☐ Specify: ____________________

Interviewer: IF THE REASONS CITED BY THE INTERVIEWEE REFER TO BLOCK B ABOVE, SKIP TO QUESTION 39 ➡

C. REASONS RELATED TO THE HEALTH FACILITY'S LOGISTICS & ORGANIZATION

1. There were no vaccines ☐
2. There were no syringes, or other vaccination supplies were missing ☐
3. It is not a vaccination day in this health facility ☐
4. The vaccination area was closed ☐
5. The person in charge of vaccinations was not there ☐
6. There would have been a long wait ☐
7. The staff treated us badly ☐
8. The hours for vaccination are limited ☐
9. Other ☐ Specify: ____________________

Interviewer: IF THE REASONS CITED BY THE INTERVIEWEE REFER TO BLOCK C ABOVE, SKIP TO QUESTION 39 ➡

---

30. How long did you wait today for your child to be vaccinated? Hours: _____ Minutes: ______

QUALITY OF THE VACCINATION SERVICE

31. Did they tell you today what vaccines they gave the child?
    1. Yes ☐
    2. No ☐

32. Did they tell you today the date of the next vaccination appointment and write it in the vaccination card/health passport?
    1. Yes ☐
    2. No ☐

33. Did you receive information today on the reactions or side effects that can occur following vaccination?
    1. Yes ☐
    2. No ☐ ➡ **SKIP TO QUESTION 36**

34. If so, what did they tell you ____

35. Did you receive information today on what you should do if the child has reactions or side affects to the vaccines?
    1. Yes ☐
    2. No ☐

36. Are you satisfied with the service provided today?

1. Yes ☐
2. No ☐ ➡ **SKIP TO QUESTION 38**

37. Why?

1. Immediate attention ☐ ➡ **SKIP TO QUESTION 39**
2. Friendly treatment by staff ☐ ➡ **SKIP TO QUESTION 39**
3. No charge for service ☐ ➡ **SKIP TO QUESTION 39**
4. Other ☐ Specify: __________ ➡ **SKIP TO QUESTION 39**

38. Why were you NOT satisfied?

1. Had to wait a long time ☐
2. The staff was discourteous ☐
3. The language that the health workers use is not clear ☐
4. They did not explain what vaccines they had given the child ☐
5. Other ☐ Specify: __________

39. Have you ever been asked to pay for vaccines given to a child?

1. Yes ☐
2. No ☐ ➡ **SKIP TO QUESTION 41**

40. What type of health facility asked you to pay?

1. Public ☐
2. Private ☐
3. DK ☐

REASONS TO VACCINATE CHILDREN

41. Could you tell me the purpose of vaccines? (CHECK ALL THAT APPLY) **Please DO NOT read out the options**

1. To prevent diseases ☐
2. So children will grow up health ☐
3. To cure/heal diseases ☐
4. They don't do any good ☐
5. Not sure what they're for ☐
6. Other ☐ Specify: __________

42. What diseases do vaccines prevent? (CHECK ALL THAT APPLY) **Please DO NOT read out the options**

1. Tuberculosis ☐
2. Hepatitis ☐
3. Poliomyelitis or polio ☐
4. Diphtheria ☐
5. Whooping cough or pertussis ☐
6. Tetanus ☐
7. Pneumonia ☐
8. Meningitis ☐
9. Diarrhea ☐
10. Influenza ☐
11. Measles ☐
12. Rubella ☐
13. Mumps ☐

14. Yellow fever ☐
15. Cancer ☐ Specify: __________
16. Other ☐ Specify: __________
17. None ☐
18. DK ☐

43. Do you think your child could get diseases if you don't vaccinate him/her?

1. Yes ☐
2. No ☐
3. DK ☐

44. What suggestions do you have to improve vaccination services? **(CHECK ALL THAT APPLY)**

1. There should be more vaccination personnel ☐
2. There should be less of a wait ☐
3. Hours and days when vaccinations are available should not be limited ☐
4. Vaccination should remain free ☐
5. The treatment of the public, and of the children being vaccinated, should be friendlier ☐
6. The health center should always have vaccines ☐
7. They should provide information on the vaccines that are being given, on the diseases that they prevent, and on the reactions that they produce. ☐
8. Other ☐ Specify: ___________________
9. None ☐
10. DK ☐

Interviewer: Thank the interviewee and note the time when the interview was concluded. Read the following statement: "Remember that vaccination is a right for all people. Demand this right and remember to bring your child's vaccination card to the health facility each time you visit the centre for any reason.

Ending time: Hour: |___|___| Minutes: |___|___|

Interviewer's remarks: ____
____
____

Supervisor: Please check the completed form for accuracy and completeness

1. Form is complete and accurate (skip patterns adequately observed) ☐
2. Errors or inconsistencies on the form ☐

Supervisor's remarks: ____
____
____

Supervisor's full name: ____
Supervisor's signature: ____
