# Supplementary material for: Can vaccination coverage be improved by reducing missed opportunities for vaccination? Findings from assessments in Chad and Malawi using the new WHO methodology
Source: PLoS One. 2019 Jan 24;14(1):e0210648. doi: 10.1371/journal.pone.0210648 (PMC6345496; doi:10.1371/journal.pone.0210648)
Supplement: S4 File — (PDF) [file pone.0210648.s004.pdf]

ANNEX 5: HEALTH WORKER SURVEY

The Ministry of Health, in collaboration with the African Regional Office of the World Health Organization, wishes to strengthen the technical skills of all health workers, especially those who provide immunization services. This questionnaire has been designed to identify future training topics in immunization for all health workers. Your collaboration is greatly appreciated.
Your name is not included in this questionnaire and your participation is voluntary.

If you decide to participate, please use a pen to mark answers that in your opinion respond appropriately to the question or problem presented.
Responses will not serve as the basis for any evaluation of your professional abilities.
Read each section of the questionnaire carefully, and please do not leave any questions blank.

Questionnaire Serial Number ☐☐☐

Date of interview

Day |__|__| Month |___|___| Year |___|___|

A. Type of service

1. Public/Government service ☐
2. Private ☐
3. NGO ☐
4. Faith-based organization
5. Other ☐ Specify: _______________________________

B. Health facility type

1. Hospital ☐
2. Clinic * ☐
3. Health center ☐

* Includes other types of primary care facilities such as doctors' offices, dispensaries, limited-service clinics, and vaccination posts.

GEOGRAPHICAL LOCATION

State/Province/Region ___________________
District ___________________
Name of health facility _______________________________________

I. BACKGROUND INFORMATION

PLEASE MARK THE CORRECT ANSWER IN THE FOLLOWING SECTION:

1. Sex ☐1. Male ☐2. Female

2. Age |___|___ years

3. Professional training
   - 3.1 Clinician ☐
   - 3.2 Assistant Environmental Health Officer) ☐
   - 3.3 Nurse or midwife ☐
   - 3.4 Health surveillance officer (HSA) ☐
   - 3.5 Other ☐ Specify: _____________________

4. Area in which you work
 4.1 Outpatient visits, Emergency room ☐
 4.2 Inpatient department ☐
 4.3 Preventive medicine, Epidemiology, or Immunization ☐

5. Time in this post |___|___ years |___|___| months

6. Have you received training or participated in courses on vaccination or vaccine-preventable diseases?
 1. Yes ☐
 2. No ☐ ➡ SKIP TO QUESTION 8

7. If so, when were you last trained?
 1. <1 year ago ☐
 2. 1-2 years ago ☐
 3. 2-3 years ago ☐
 4. >4 years ago ☐

8. Do you have opportunities for clinical or academic trainings as part of your job?
 1. Yes ☐
 2. No ☐ ➡ SKIP TO QUESTION 10

9. If so, has a topic related to vaccination or vaccine-preventable diseases (VPDs) been presented in the last 12 months?
 1. Yes ☐
 2. No ☐

II. KNOWLEDGE OF VACCINATION

FOR QUESTIONS 10 - 13, PLEASE CHECK ALL CORRECT OPTIONS

10. Vaccines that healthy children should receive include: (PLEASE CHECK ALL THAT APPLY)
 1. BCG ☐
 2. Measles ☐
 3. DTP/Pentavalent ☐

11. Diseases prevented by the pentavalent vaccine include:
 1.Diphtheria, whooping cough, tetanus, Hepatitis B, pneumonia and meningitis caused by Haemophilus influenzae type b ☐
 2. Diphtheria, whooping cough, tetanus, Hepatitis B, and influenza ☐
 3. Poliomyelitis, diphtheria, whooping cough, tetanus, pneumonia, and meningitis caused by pneuomococcus ☐
 4. Diphtheria, whooping cough, tetanus, poliomyelitis, pneumonia, and meningitis caused by Haemophilus influenzae type b ☐
 5. None of the above ☐

12. Contraindications for being vaccinated against poliovirus include:
 1. Breastfeeding ☐
 2. Axillary or rectal temperature of 37.5 C ☐
 3. Mild malnutrition ☐
 4. Mild diarrhea ☐
 5. None of the above ☐

14. Age at which the following vaccines should be administered. **Please write in the blank column** of the first box the number (e.g. 5)that corresponds to the correct answer in the second box

		Blank column
A	DPT/Pentavalent	
B	PCV	
C	BCG	
D	OPV	
E	1st dose of Measles	

1	At birth, and 6, 10 and 14 weeks
2	6, 10, 14 weeks
3	9 months
4	6, 10, 14 weeks
5	At birth

FOR QUESTIONS 15 - 17, PLEASE MARK ONLY ONE CORRECT ANSWER

15. Vaccines used to prevent penumonia in children aged <2 years. **Mark only one response:**

1. Influenza, Hepatitis B, and pneumococcal conjugate vaccine ☐
2. BCG, pentavalent, and Hepatitis B ☐
3. Rotavirus, pentavalent, and pneumococcal conjugate vaccine ☐
4. Pentavalent, influenza, and pneumococcal conjugate vaccine ☐
5. None of the above ☐

16. Vaccine-preventable diseases (VPDs) in the process of eradication or elimination include:
Mark only one response:

1. Chickenpox ☐
2. Whooping cough ☐
3. Poliomyelitis ☐
4. Measles, rubella, and congenital rubella syndrome ☐
5. Only options 3 and 4 are correct ☐

17. Contradications for any vaccine:

1. Local reaction to previous dose ☐
2. Light fever ☐
3. Seizures under medical treatment ☐
4. Pneumonia or other serious diseases ☐
5. None of the above ☐

18. Do you believe that in some situations a person vaccinated against a disease could contract that same disease years later?

1. Yes ☐
2. No ☐

Explain your response: ______________________________________

19. For the following list of vaccines, place a check (X) in the column marked **AT** if the vaccine is attenuated or a check on the column marked **IN** if the vaccine is inactivated, recombinant, or fractional.

	Vaccines	AT	IN
1	BCG		
2	DPT		
3	Measles		
4	PCV		
5	OPV		
6	Haemophilus influenzae type b (Hib)		
7	Hepatitis		

III. ATTITUDES

FOR QUESTIONS 20-23, PLEASE MARK ONLY ONE CORRECT ANSWER.

20. From day to day, who should evaluate the vaccination status of children, review vaccinaiton cards/health passports, and ensure that children are up to date according to the national schedule?
    1. The child's parents ☐
    2. The health worker responsible for immunization ☐
    3. Physicians in external consultations, inpatient services, and emergency room ☐
    4. All of the above ☐
    5. Only options 1 and 2 are correct ☐

21. In which of the following situations should you inquire about the doses that children have received and those that are missing according to their age?
    1. Child's wellness visit ☐
    2. Consultation for any illness ☐
    3. When a child is accompanying a caregiver during a prenatal check-up ☐
    4. When a child is accompanying a caregiver visiting a health care facility for any reason ☐
    5. All of the above ☐

22. Why do you think that some children are not up to date on their vaccination?
    1. Parents' negative beliefs related to vaccination ☐
    2. Hours of vaccination incompatible with parents' busy lives ☐
    3. Physicians, nurses, and health workers do not ask about children's vaccination schedules ☐
    4. Physicians, nurses, and health workers do not review children's vaccination cards/health passports ☐
    5. False contraindications for vaccination by health workers ☐
    6. Distance from vaccination site ☐
    7. All of the above ☐

23. Do you believe that the vaccines administered in private practice vary in quality from those provided by the Ministry of Health?
    1. Yes ☐
    2. No ☐
    3. Don't know ☐

24. Please explain your response in Question 23: ____

FOR QUESTIONS 25-28, PLEASE INDICATE IF YOU AGREE OR DISAGREE WITH THE FOLLOWING STATEMENTS:

25. My knowledge of vaccines and vaccination is insufficient or outdated:
    1. Agree ☐
    2. Disagree ☐
26. The knowledge I have about vaccination and the Expanded Program on Immunization (EPI) is sufficient to meet the needs of the institution:
    1. Agree ☐
    2. Disagree ☐

27. I am very concerned about, and fear, adverse reactions from vaccines.
    1. Agree ☐
    2. Disagree ☐

28. Completing nominal vaccine registries (books/notebooks) delays the timely vaccination of children
    1. Agree ☐
    2. Disagree ☐

IV. PRACTICES

IV.I DECISION MAKING IN DAILY PRACTICE

THIS SECTION IS FOR ALL HEALTH CARE PROFESSIONALS

FOR QUESTIONS 29-32, MARK THE ONE CORRECT ANSWER

29. Female infant aged 3 months with documented history of one dose of BCG and one of OPV0, both administered at birth. The mother seeks service to assess the child's growth and development. What vaccines would you give the child?
    1. None ☐
    2. Only Polio ☐
    3. Only Pentavalent/DTP ☐
    4. Measles vaccine ☐
    5. Polio, Pentavalent/DTP, Rota and PCV ☐

30. A newborn male weighing 3.2 kg with a normal vaginal delivery in a hospital. The mother is HIV negative but a carrier of Hepatitis B. What vaccines should this child receive before leaving the hospital?
    1. BCG vaccine ☐
    2. Hepatitis B vaccine ☐
    3. Pentavalent/DTP ☐
    4. None of the above ☐
    5. Only options 1 and 2 are correct ☐

31. Female infant aged 6 months with documented history of one dose of BCG, two doses of pentavalent, and two doses of polio vaccine. The last doses of vaccines were administered when the child was 4 months old. According to the mother, the child experienced fever and seizure one month ago and is now receiving medical treatment. Following EPI guidelines, what vaccines would you give her?
    1. I would not vaccinate her ☐
    2. Only polio vaccine and I would refer her to a specialis ☐
    3. PCV ☐
    4. Polio and pentavalent/DTP ☐
    5. Only measles vaccine ☐

32. What vaccines have you ever administered to an adult? (check all that apply)
    1. TT ☐
    2. Measles ☐
    3. Hepatitis B ☐
    4. Influenza ☐
    5. OPV ☐
    6. None of the above ☐

IV. PRACTICES

IV.2 IMMUNIZATION PRACTICES AND DECISION MAKING

[THIS SECTION IS ONLY FOR ALL HEALTH CARE PROFESSIONALS WHO ADMINISTER VACCINES]

33. Under what circumstances would you tell the parent what vaccines you are administering AND provide advice regarding what to do in case the child experiences an adverse reaction following immunization?
    1. Only if the vaccine administered could produce a severe reaction ☐
    2. Only when the parent or guardian requests this information ☐
    3. Never, since this information can be counterproductive and discourage participation in the immunization programme ☐
    4. Always, regardless of the vaccine used and type of reaction that might be expected ☐
    5. The probability that an adverse event related to vaccination is so low that I would rarely have to provide this information ☐

34. Today, you vaccinate a female child aged 2 months with the first doses of pentavalent/DTP, and polio vaccines. After telling her parents which vaccines she received, what other information and recommendations would you provide her caregivers?
    1. The child may experience a bit of fever, diarrhea, or discomfort following vaccination ☐
    2. The symptoms above generally do not require treatment; however, in the case of fever, the child should be lightly dressed and should not stop breastfeeding under any circumstances ☐
    3. The parent should return to the health center if these symptoms persist so that the child may be seen by a doctor ☐
    4. All of the above ☐
    5. None of the above ☐

35. What should be done if you notice that there are children with delayed vaccination schedules in the nominal registeries of a health center?
    1. Make a weekly list of children with incomplete schedules ☐
    2. Contact parents or guardians by telephone, email, or any other means of communication to remind them to vaccinate their children ☐
    3. Make home visits to encourage the family to complete the child's vaccination schedule and administer missing doses while there ☐
    4. All of the above ☐
    5. None of the above ☐

36. What could be done to follow up on vaccination of children after hospitalization or outpatient treatment for a chronic condition?
    1. Coordinate with clinical areas, inpatient and emergency departments in hospitals, so that they can review the child's vaccination card/health passport ☐
    2. Send patients whose physicians consider them eligible for vaccination to the immunization department so that they can be vaccinated before leaving the hospital ☐
    3. In hospitals, a health worker in the immunization department could

visit inpatient departments to review to review the medical records of children who will be discharged that day, thereby identifying children to start or complete the vaccination schedule ☐
4. All of the above ☐
5. None of the above ☐

37. At 8:00 am, you prepare a vaccination cold box for the morning shift at the health facility. Following proper cold chain guidelines for cold boxes, you place two vials of 10 doses of measles vaccine in the cold boxes. At 3:00 pm, a mother requests that her 13 month old child receive one dose of measles vaccine. After reviewing the child's card, you determine that the child has not yet received measles vaccine but that she has otherwise received all vaccines for children aged < 1 year. Additionally, the child has no contraindications. Only two doses from the first vial have been administered since 8:30am, when the first dose was administered. Which of the two vaccine vials in the cold box would you use to vaccinate the child?
1. I would use the open vial to prevent vaccine wastage ☐
2. I would tell the mother to return the next day, since I cannot open a new vaccine vial and there are no more children to vaccinate ☐
3. I would open the second vial of measles vaccine to immunize the girl ☐
4. I would recommend that the mother take the child to another health center to be vaccinated ☐
5. None of the above ☐

38a. What instructions do you usually give to caregivers the first time you issue them a new vaccination card/health passport? (PLEASE CHECK ALL THAT APPLY)
1. Keep the card safe ☐
2. Bring this card to all visits to the health facility ☐
3. Bring this card only when you come for vaccinations ☐
4. No instructions are given ☐
5. Others: Specify: ____________________________ ☐

38b. What do you do for a caregiver that forgot the vaccination card/health passport at home:
1. I do not vaccinate the child and ask mother to return with card next time ☐
2. I issue a new card, vaccinate and record today's vaccinations ☐
3. I issue a new card, vaccinate and record old vaccinations from the register ☐
4. I issue a temporary card, vaccinate and ask them to bring the old card for the next visit ☐
5. Vaccinate without replacing card, document in register only ☐
6. Other: Specify: ____________________________ ☐

38c. If a woman reports that the child's card has been lost or damaged, what do you usually do?
1. I issue a new card and record all future vaccines in the new card ☐
2. I issue a new card and transcribe all previous vaccines from register ☐
3. I issue a new card and ask woman to tell me of all previous vaccinations so I can write them down ☐
4. Vaccinate without replacing card, document in register only ☐
5. Other: Specify: ____________________________ ☐

39. In the following list of resources and procedures for the cold chain, place a check mark (X) in correct statement if it is applicable to refrigerators, cold boxes, or both

No.	Reources and procedures	Refrigerator	Cold boxes
1	Storage at 2-8 C		
2	Conditioned ice packs		
3	15 cm away from the wall		
4	Temperature log book		

IN THE FOLLOWING SECTION, INDICATE WITH A CHECK MARK WHETHER YOU AGREE WITH THE FOLLOWING STATEMENTS:

40. There is sufficient staff offering immunization services at this health facility
    1. Agree ☐
    2. Disagree ☐

41. Today, I have enough vials of all vaccines for patients who seek immunization services
    1. Agree ☐
    2. Disagree ☐

42. Today, I have all the materials that I need to vaccinate patients who seek immunization (including syringes, recording sheets, vaccination cards/health passports, and other materials)
    1. Agree ☐
    2. Disagree ☐

43. When the professional in charge of vaccination is unavoidably absent, a health care professional is available to replace him or her
    1. Agree ☐
    2. Disagree ☐

ADDITIONAL COMMENTS:

____

____

____

____

____

Thank you for your time and have a wonderful day!
